# Benzoxazinoids stimulate chemotaxis and act as a signaling molecule in *Azospirillum brasilense* Ab-V5, while showing minor effects on *Pseudomonas protegens* Pf-5

**DOI:** 10.1128/mbio.01414-25

**Published:** 2025-07-31

**Authors:** Jeroen Baatsen, Guilherme K. Hosaka, Mateus Mondin, João L. Azevedo, Mariangela Hungria, Maria C. Quecine

**Affiliations:** 1 Laboratory of Genetics of Microorganisms “Prof. Joao Lucio de Azevedo”, Department of Genetics, University of Sao Paulo 28133, São Paulo, Brazil; 2 CYNGELA – Cytogenetics and Epigenetics Laboratory, Department of Genetics, Luiz de Queiroz College of Agriculture, Universidade of Sao Paulo 28133, São Paulo, Brazil; 3 Embrapa Soja 593385 , Londrina, Brazil; The Pennsylvania State University, University Park, Pennsylvania, USA

**Keywords:** PGPB MBOA, chemotaxis, biofilms, peroxidases, symbiosis, transcriptomics, SEM, root colonization

## Abstract

Root colonization by plant growth-promoting bacteria (PGPB) involves recruiting beneficial partners from the rhizosphere. Among well-studied PGPB, *Azospirillum brazilense* Ab-V5 and *Pseudomonas protegens* Pf-5 are two well-known bacterial strains renowned for their growth-enhancing capacity and extensively used as bio-inputs. Many cereals, such as maize, produce indole-derived benzoxazinoids (BXs), specialized metabolites that shape root-associated microbiomes to promote colonization by plant-growth-promoting bacteria (PGPB). Although the mechanisms by which BXs recruit PGPB remain unclear, we hypothesize that BXs directly facilitate root colonization by favoring bacteria adapted to these metabolites in the soil environment. In this study, we investigated the impact of the relatively stable lactam BX-derivative, 6-methoxy-2-benzoxazolinone (MBOA), on two PGPB strains: *Azospirillum brasilense* Ab-V5 and *Pseudomonas protegens* Pf-5. Transcriptomic analysis revealed that MBOA had minimal effects on Pf-5, but triggered extensive gene expression changes in Ab-V5, particularly in pathways related to energy metabolism, chemotaxis, and biofilm formation. Subsequent assays confirmed that MBOA acts as a chemoattractant for Ab-V5 and, at moderate concentrations, enhances both biofilm formation and colonization of *Arabidopsis* roots. We propose that the chemotactic property of MBOA on Ab-V5 can enhance its establishment in the rhizosphere and that this metabolite can trigger the metabolic transition required for root colonization.

**IMPORTANCE:**

In this paper, we studied the impact of benzoxaziniods on root colonization mechanisms of two potent plant-growth- promoting bacterial strains. We explored these mechanisms by an RNA sequencing experiment and by microscopy. The paper highlights how biofilm is particularly affected and reports on chemotactic responses. Most of the results we obtained we could validate with phenotypic assays. We show that benzoxazinoids, produced by many cereals, profoundly affect bacterial behavior related to plant-bacterial interactions. The bacteria in this study are known for their ecological roles in the soil, being either in plant protection or as biofertilizers. Thus, this work holds significant socio-economic value for society.

## INTRODUCTION

Benzoxazinoids (BX) are specialized metabolites produced by many grasses including rye (*Secale cereale* L.), wheat (*Triticum aestivum* L.), and maize (*Zea mays* L.) (1). Released in the soil by roots, they strongly affect the composition of the rhizomicrobiome, thereby stimulating a vast array of positive features attributed by plant growth-promoting bacteria (PGPB), ranging from nutrient acquisition to plant defense (1–10) *Azospirillum brasilense* is among the best-studied PGPB, reportedly granting growth-promoting properties predominantly by the production of plant hormones (11–13) and biological nitrogen fixation (BNF) (14). *A. brasilense* exhibits extraordinary genome plasticity characterized by numerous repetitive sequences and origins of replications (15), and its genome harbors several genes associated with rhizosphere adaptation (15). Biosynthesis of plant hormones by *A. brasilense*, including strain Ab-V5, stimulates the development of lateral roots and root hairs (16, 17) leading to improved water and mineral uptake (18, 19). In addition to yield increase of maize, wheat, and pastures with brachiarias (*Urochloa* spp.) (20, 21), inoculation with Ab-V5 confers stress tolerance by stimulation of jasmonic acid (JA) and salicylic acid (SA) pathways and peroxidase activity (22, 23). For these reasons, *A. brasilense* strain Ab-V5 is widely used in commercial inoculants in Brazil (24).

Regarding biocontrol, the PGPB *Pseudomonas protegens* strain Pf-5 (formerly *Pseudomonas fluorescens* Pf-5), is well known for producing a wide array of antimicrobial specialized metabolites, notably pyoluterin and 2,4-diacetylphloroglucinol, two potent antimicrobial components (25). Therefore, *P. protegens* Pf-5 is of special interest as a biocontrol strain and for conferring disease tolerance (26–30). The *P. protegens* species colonizes a wide variety of plant hosts (31–33) demonstrating a remarkable metabolic flexibility, with some strains even possessing the capacity to use insects as vectors for dispersal (34, 35).

Recruitment of bacteria is a crucial step in root colonization and is key for the introduction of superior PGPB strains. Chemoattraction of PGPB toward root exudates has been extensively studied (6, 36–39) and is an inextricable part of recruitment in the rhizosphere. Conversely, specific studies on chemotaxis to BX-derivatives are limited to a single study by Neal and collaborators on chemoattraction of *P. putida* to 2,4-dihydroxy-7-methoxy-1,4-benzoxazin-3-one (DIMBOA) (6). In the soil, BXs degrade spontaneously, primarily converting into 6-methoxy-2-benzoxazolinone (MBOA) (40), which exerts a lasting influence on microbiome structuring, persisting into the next generation of maize plants (7). Interestingly, once root colonization has been initiated, *A. brasilense* and *P. fluorescens* establish a positive feedback loop by stimulating BX metabolism of the plant upon root colonization (41, 42). Consequently, the exudation of BX derivatives potentially influences the chemoattraction of PGPB, promoting rhizospheric colonization. This process, in turn, stimulates plant BX metabolism, demonstrating a sophisticated plant-bacteria signaling dialog.

Despite substantial research done on the impact of BXs on microbial structuring (7–10, 43–45), how specific mechanisms are affected by BX in individual PGPB is poorly understood. Thus, both Ab-V5 and Pf-5 serve as compelling study subjects for analyzing the influence of MBOA on their respective transcriptomes to clarify the intricate mechanisms underlying plant-soil feedback. In addition, given their natural occurrence being either in association with BX-producing cereals or in predominantly BX-free soil environments, respectively, Ab-V5 and Pf-5 make a keen comparison and may provide insights on how bacteria are adapted to MBOA exposure.

Given the positive influence of BXs on the whole root microbiome (7–10, 43–45), we hypothesized that root colonization mechanisms of individual PGPB are likely to be manipulated. Therefore, we carried out RNA sequencing of Ab-V5 and Pf-5 RNA extracts to unravel what cell-physiological processes are influenced by MBOA treatment. We found evidence on a molecular level showing how MBOA affects bacterial behavior and colonization mechanisms of Ab-V5 and Pf-5 validated with microbiological, biochemical, and microscopic assays. To the best of our knowledge, this study represents the first transcriptomic analysis conducted on individual PGPB to investigate the direct impact of BXs on RNA profiles.

## MATERIALS AND METHODS

### Bacterial strain and growth conditions

For this study, we used the strain *A. brasilense* Ab-V5 (20) which was isolated from the maize (*Zea mays L*.) rhizosphere and *P. protegens* strain Pf-5 originally isolated from the rhizosphere of cotton (*Gossypium hirsutum* L) seedlings (26). Strain Ab-V5 (CNPSo 2083) was provided from the “Diazotrophic and Plant Growth-Promoting Bacteria Culture Collection” (WFCC collection #1213, WDCM Collection #1054), Londrina-PR, Brazil, and strain Pf-5 was provided by Dra. Joyce E. Loper from Horticultural Crops Research Laboratory, United States Department of Agriculture, Agricultural Research Service. Bacterial cultures were stored in 20% glycerol at −80°C. At the onset of the experiment, Ab-V5 precultures were grown in DYGS liquid medium (46) and Pf-5 in Luria-Bertani (LB) medium (47) shaking at 28°C, replicated from bacterial stock until early log-phase, and diluted to optical density at 600 nm (OD_600_) of 0.05. From a start OD_600_ of 0.05, the bacteria were used in *in vitro* experiments or for infecting plant roots as described in item 2.7.

### Effect of MBOA on bacterial growth

First, the tolerance of PGPB to MBOA (cat. no. 532-91-2, Sigma-Aldrich, Saint Louis, USA) was assessed by obtaining growth curves from bacteria grown in DYGS and LB liquid culture medium supplemented with increasing concentrations of MBOA. To that end, pre-cultures were freshly prepared at the onset of the experiment from bacterial stocks and grown until the early logarithmic phase in the PGPBs’ respective growth medium. The OD_600_ was adjusted to 0.05 in 100 mL Erlenmeyer flasks containing 20 mL liquid growth medium amended with 0.05 mM, or 0.50 mM MBOA from a 100 mM MBOA stock solution prepared in acetone. The control treatment contained 0.5% acetone which equals the amount of MBOA solution in the other treatments. Over the time course of 24 h, cultures were grown at 120 rpm and 28°C, and every 3 h, after 16 h and 24 h, the OD_600_ of 1 mL of each culture was measured by spectrophotometry with a Genesys 50 UV-Vis spectrophotometer (Thermo Scientific, Massachusetts), while the other flasks remained incubated under constant agitation. The experiment was performed with four biological replicates per treatment and was carried out twice.

To study the impact of MBOA on bacterial population dynamics after longer incubation times, Ab-V5 and Pf-5 start cultures were grown from a preculture; diluted until OD_600_ of 0.05 and treated with 0.00 mM containing 0.5% acetone, 0.05 mM MBOA and 0.50 mM MBOA in triplicate. At the timepoints 0 h, 24 h, 48 h, and 72 h, cultures were diluted and plated out to determine the colony-forming units (CFU) by enumeration on solid bacterial culture medium.

### RNA extraction and sequencing

Based on the results of the growth curve, each bacterial inoculum was grown in 0.00 mM, 0.05 mM, and 0.50 mM MBOA for 72 h at 28°C statically in order to promote biofilm formation at the air-liquid interface. Each treatment was performed with six biological replicates. RNA of bacterial cultures was stabilized by adding two times the culture volume of RNA protect bacterial reagent (Qiagen, Venlo, Netherlands), directly into 15 mL glass tubes containing 1 mL of bacterial cultures. RNA was isolated using an RNeasy RNA purification kit (Qiagen, Venlo, Netherlands) according to the manufacturer’s instructions, including a cell lysis step with 15 mg mL^−1^ lysozyme (cat. no. L6876, Sigma-Aldrich, Saint Louis, USA) and 10 mg mL^−1^ proteinase K (cat. no. RP103B, Qiagen, Venlo, Netherlands) in TE buffer of pH 8 for 10 min at room temperature. Additionally, we performed an on-column DNA digestion step with an RNase-free DNase set (Qiagen, Venlo, Netherlands). RNA was eluted in two steps with 50 µL RNase-free water in RNase-free microcentrifuge tubes and stored at −80°C. Quality control of the samples was carried out by an Agilent 2100 Bioanalyzer (Agilent, Barueri, Brazil), to select the three best biological repeats per treatment for cDNA library preparation with Illumina Stranded Total RNA prep, and ribosomal depletion with Ribo-Zero plus (Illumina, San Diego, USA). Sequencing of the samples was carried out by an Illumina NextSeq 550 system (Illumina, San Diego, USA) with a read depth of on average 13 million clusters or 26 million paired-end reads at NGS Soluções Genômicas (Piracicaba, Brazil).

### RNAseq data analysis

Initially, the raw read quality was determined using FastQC v0.12.0 (48), a commonly used tool for assessing the quality of data generated by RNA sequencing (RNA-seq). After assessing sample quality, Trimmomatic v0.39 (49) was employed to filter out low-quality reads and remaining sequencing adapters applying a cut-off for Phred quality scores below 25 and removal of Nextera – PE adapters. The filtering of rRNAs from the samples was carried out using RiboDetector v0.2.7 (50), a specialized tool designed to identify ribosomal RNA (rRNA) sequences and filter them from RNAseq data which can constitute a significant proportion of the reads obtained during RNAseq and complicate the analysis of gene expression by misalignment. From the Ab-V5 reads, we aligned the trimmed and filtered reads with STAR v2.7.10 (51) to the Ab-V5 genome (GenBank accession: GCA_002940725.1) and Pf-5 reads were aligned to the Pf-5 genome (Genbank accession: CP000076), while gene quantification was carried out with HTSeq-count v0.11.1 (52). The R package edgeR v4.2.0 (53) was used to filter out samples with low expression, considering genes that had at least one count per million in at least three samples. The same package was used to normalize the data and analyze it for differential expression among treatments. The differential expression analysis used the 0.00 mM MBOA treatment as a reference level. For each comparison, we tested the null hypothesis H0:LogFC=0 at a significance level of *P* = 0.05. In this context, LogFC represents the logarithm of the fold change expression value. For functional annotation, DIAMOND v2.1.7 (54) was performed with the non-redundant (nr) NCBI database. Blast2GO suite (55) was used to categorize the annotated genes via DIAMOND v2.1.7 (54) into functional Gene Ontology (GO) terms. Non-annotated and hypothetical proteins we classified as “unknown gene function” and were not further considered in the analysis.

### Effect of MBOA on bacterial chemotaxis

Chemotaxis responses of strains Ab-V5 and Pf-5 were assessed by a modified capillary assay (56). Briefly, sterile syringes of 0.5 mL with needles of 0.25 µm aperture were filled with MBOA or a 0.5% acetone equivalent in phosphate-buffered saline (PBS, 8 g L^−1^ NCL, 0.2 g L^−1^ KCl, 1.44 g L^−1^ Na_2_HPO_4_, 0.24 g L^−1^ KH_2_PO_4_) of pH 7.4. The syringes were inserted into 15 mL Falcon tubes containing 5 mL of washed bacteria in PBS with an OD_600_ of 0.05. After incubation at room temperature for 15 min, syringes were ejected and 100 µL of Ab-V5 or Pf-5 was directly plated on 15% agar DYGS or LB plates, respectively, rendering five plates per syringe. Colony-forming units (CFU) were counted digitally using ImageJ software (Scion Corporation, Maryland). For every treatment, at least four biological replicates were used, and the experiment was carried out three times.

For validating the results from the capillary assay, chemotaxis and motility were assessed with a swim plate assay and gradient plate assay based on Mukherjee et al. 2016 (57). In short, liquid precultures were grown in liquid medium until an OD_600_ of around 0.4 was reached, adjusted to OD_600_ 0.4, and the bacterial cultures were washed three times in chemotaxis buffer (50 mM K_2_HPO_4_, 10 µM EDTA, 0.05% glycerol, pH 7). In the meanwhile, for the swim plate assay, either malate-salt medium (MSM) or M9 minimal medium plates for Ab-V5 or Pf-5, respectively, containing 0.3% agar, 1 mM glycerol, and amended with 0.00 mM, 0.05 mM, or 0.50 mM MBOA, were prepared. Fifty microliters of Ab-V5 culture was applied on the swim plate and incubated for 48 h before recordings. Ten microliters of OD_600_ 0.4 of Pf-5 culture was applied and recorded after 8 h of incubation. For the gradient assay, the 0.3% agar plates did not contain MBOA but were provided with a 1.5% agar plug containing 0.00 mM, 0.05 mM or 0.50 mM MBOA, placed 2 cm from either 50 µL of Ab-V5 or 10 µL of Pf-5 cultures.

### Effect of MBOA on bacterial biofilm

To determine the influence of MBOA on the production of biofilm by Ab-V5 and Pf-5, a microtiter plate biofilm assay was carried out, using sterile polystyrene 96-well plates as described in Merritt et al. (58) Briefly, overnight bacterial cultures were diluted until OD_600_ of 0.05 (approximately 10^8^ bacteria) in DYGS for Ab-V5 or in liquid LB for Pf-5 and supplemented with 0.00 mM, 0.05 mM, or 0.50 mM MBOA. Microtiter plates were filled with 100 µL of bacterial culture using at least eight replicates of each treatment including non-inoculated controls for 72, 96, 120, and 144 h of stationary incubation at 28°C. The microtiter plates were then rinsed to remove planktonic bacteria. Biofilm was stained with 125 µL 0.01% (wt/vol) crystal violet per well for 20 min. After removal of the unbound crystal violet, each well was filled with 150 µL of 100% ethanol for 15 min which was then transferred to an optically clear microtiter plate and analyzed with a Multiskan FC Microplate Photometer (Thermo Scientific, Massachusetts) at OD_590_.

### Plant growth conditions

Seeds of *Arabidopsis thaliana* Col-0 were surface sterilized by suspending the seeds in 70% ethanol for 2 min and in 50% hypochlorite for 10 min followed by rinsing three times with sterile deionized water. Sterile seeds were placed on half strength Murashige and Skoog medium (½ MS) (cat. no. M5519, Sigma-Aldrich, Saint Louis, USA) plates containing 0.8% agar and 1% sucrose. After an incubation period in the dark for 3 days, plates were placed vertically in an incubation room at 22°C under a 16 h/8 h light/dark regime in order for the roots to grow on the surface of the ½ MS agar plates. After 2 weeks of incubation, seedlings were inoculated with an MBOA-treated bacterial culture of OD_600_ of 0.05 and were tested for peroxidase activity (59) of Ab-V5 to the root surface or analyzed by microscopy, as described in the Section “Epifluorescence Microscopy and Scanning Electron Microscopy.”

### Epifluorescence microscopy and scanning electron microscopy

Two-week-old *A. thaliana* seedlings were inoculated 96 h before analysis with washed Ab-V5 or Pf-5 cultures with OD_600_ of 0.05 with or without addition of 0.05 mM MBOA, or with the same amount of sterile deionized water as the control treatment. Prior to epifluorescence microscopy, *A. thaliana* seedlings were supplemented with 3 mL of 2 µg mL^−1^ NileRed solution (9-diethylamino-5H-benzo[a]phenoxazine-5-one) (cat. no. 7385-67-3, Sigma-Aldrich; Saint Louis, USA); incubated at room temperature for 1 h at 120 rpm; rinsed with sterile Milli-Q (Merck, Germany) purified water; and carefully transferred on microscopic slides and sealed. Nile Red is a lipophilic stain that has an emission wave length of around 540 nm when bound to neutral lipids and around 650 nm when bound to polar lipids (60–62). Epifluorescent microscopic analysis was carried out using an Axiophot II microscope (Zeiss, Germany) with magnifications within the range of 100–400 times, and with the following excitation (Ex) and emission (Em) filter settings: Ex 365–Em 397 (blue channel), Ex 450–Em 515 (green channel), Ex 546–Em 590 (red channel). Images were captured through a PCO CCD camera operated by ISIS Metasystems.

Preparation of samples for scanning electron microscopy (SEM) included a primary fixation step with 2.5% glutaraldehyde in 0.2 M cacodylate; secondary fixation with 2% osmium tetroxide overnight; dehydration in a series of ethanol solutions in increasing concentration (10%, 20%, 30%, 50%, 70% 10 min per step and three times in 100% ethanol); drying with a Baltec EM CPD 300 (Baltec, Lichtenstein) critical point drying machine and gold coating with a Baltec SCD 050 (Baltec, Lichtenstein) gold coater. After mounting the samples on stubs, they were analyzed by a JEOL JSM-IT300LV (JEOL, Japan) located at the Phytopathology Department at ESALQ/USP (Piracicaba, Brazil) using an accelerating voltage of 20 kV and magnifications varying between 1,400 and 7,500 times, during SEM image analysis.

The microscopic analysis by SEM was carried out three times, every time analyzing three samples per treatment of one square cm of excised *A. thaliana* seedling roots from the surface of agar plates. From those samples, we scored the biofilm formation and estimated the percentage of the root surface covered by biofilm, as well as colonization patterns.

### Peroxidase assay

Peroxidase activity was evaluated by measuring the oxidation of guaiacol (2-metoxifenol) (cat. no. G5502, Sigma-Aldrich, Saint Louis, USA) by spectrophotometry based on Mika et al. (59). *A. thaliana* Col-0 seedlings grown on 0.8% agar, 1% sucrose, ½ MS medium were harvested 2 weeks after germination in samples of approximately 0.5 g. Seventy-two hours before analysis, *A. thaliana* Col-0 seedlings were inoculated with washed Ab-V5 or Pf-5 bacteria treated with 0.00 mM, or 0.50 mM, or without bacteria but with 0.50 mM MBOA as a control treatment. The seedlings were homogenized in 0.5 mL 10 mM sodium acetate of pH 5 and centrifuged for 25 min at 15,000 × *g* and 4°C, and the supernatant was used as protein extract for the peroxidase activity measurement. The measurements were started by mixing in a cuvette: 970 µL sodium acetate, 2.5 µL guaiacol 0.25% (vol/vol), 6.0 µL hydrogen peroxide 30% (wt/vol), and 20 µL protein extract. The absorbance of tetraguaiacol was then measured every 10 s along the timespan of 1 min in a Genesys 30 spectrophotometer (Thermo Scientific, Waltham, USA) at 470 nm wavelength. From the data, normalized per gram of tissue, the coefficients of the regression lines were used to calculate the peroxidase activity expressed in absorbance per minute per gram.

### Statistical analysis

Data obtained from digital analysis of pictures from plates using the ImageJ software (Scion Corporation, Maryland) for counting colony- forming units (CFU), and all other quantitative data were statistically analyzed using the R software (63). Data were first tested for normality via the Shapiro-Wilk normality test (*P* = 0.05). Normally distributed data were subjected to a one-way ANOVA and a subsequent Tukey multiple comparison of means or a Welch Two Sample *t*-test for testing two groups (*P* = 0.05). The data without normal distribution were analyzed with a Kruskal-Wallis rank sum test or Wilcoxon rank sum test with continuity correction (a.k.a. Mann–Whitney *U* test) (*P* = 0.05).

## RESULTS

### Ab-V5 is perceptible to MBOA which potentially acts as a signaling molecule, while the Pf-5 transcriptome is little affected

The impact of a low (0.05 mM) and a high dose (0.50 mM) on bacterial growth of Ab-V5 and Pf-5 liquid cultures was explored by obtaining a growth curve during the first 24 h of inoculation. (Fig. S1). Ab-V5, which in general grows slower than Pf-5, showed a stronger reduction in bacterial growth than Pf-5. From 9 h onward, in contrast to 0.05 mM, 0.50 mM significantly reduced bacterial growth compared to the control treatment, demonstrating a strong effect on bacterial behavior. However, no significant differences between MBOA and control treatments were found in the number of CFU after liquid cultures were plated 24, 48, and 72 h post-inoculation (Table S1) (Fig. S2). Therefore, we used the lowest concentration tested (0.05 mM) and a relatively high concentration of 0.50 mM MBOA in the RNAseq assay. In this way, we could discern dynamically regulated genes that mark relevant mechanisms manipulated by MBOA.

**Fig 1:**
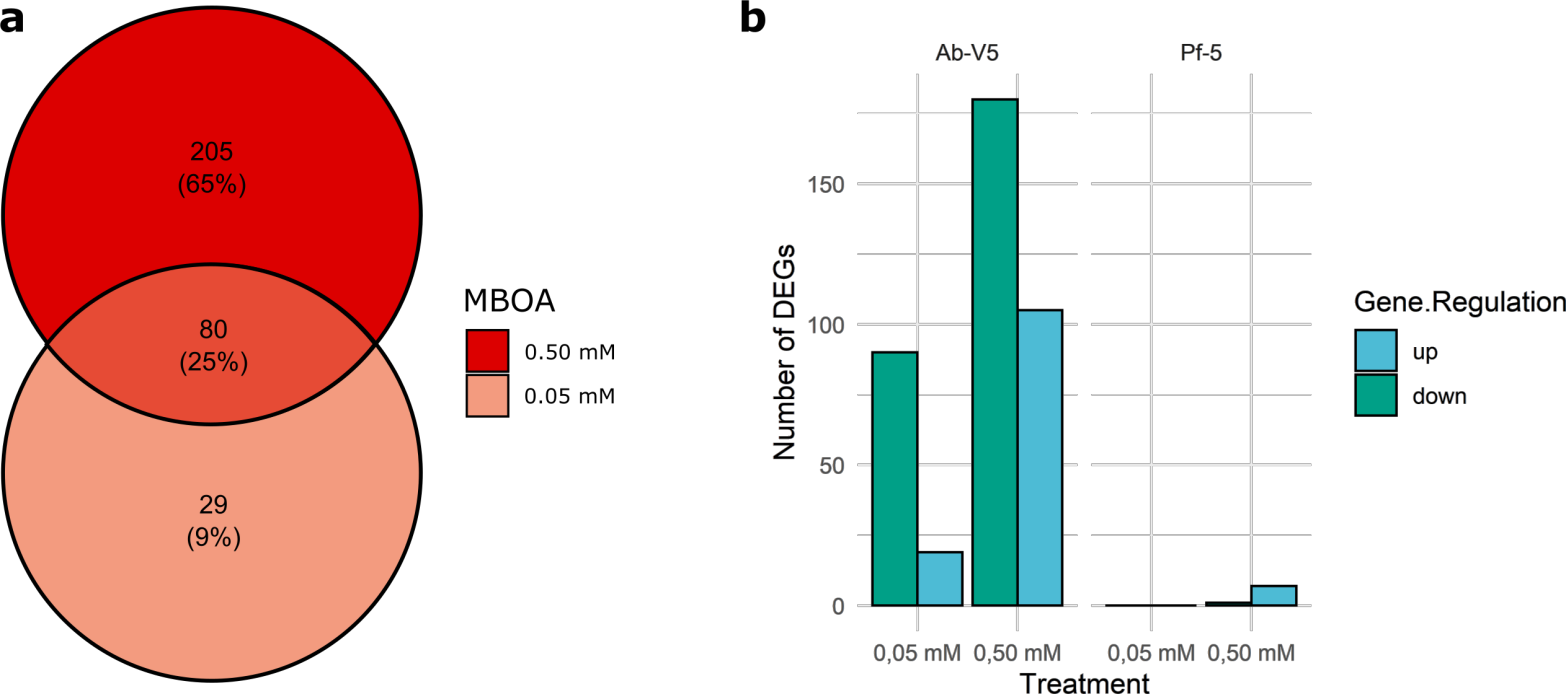
Organization of DEGs found in 0.05 and 0.50 mM MBOA treatments of *Azospirillum brasilense* Ab-V5. (a) Venn diagram showing unique and common DEGs (*P* = 0.05) among Ab-V5 treatments. (b) Bar plot displaying the number of up- and downregulated DEGs from Ab-V5 and Pf-5. No DEGs were identified from the Pf-5 transcriptome with 0.05 mM MBOA.

The transcriptome of Pf-5 underwent few alterations (Fig. 1b). Besides the 0.05 mM MBOA treatment that rendered no DEGs, 0.50 mM MBOA inflicted a significant change in the expression levels of only eight DEGs relative to the control (Table S3). Remarkably, DEGs were mainly categorized as belonging to the cellular respiration protein class and have positive logFC values, albeit with no DEGs surpassing the logFC threshold value of 2 or −2.

Introducing an environmental concentration of 0.50 mM MBOA to *A. brasilense* Ab-V5, however, resulted in a wide-scale reprogramming of metabolic regulation. The 0.50 mM MBOA treatment, which rendered 285 genes to be differentially expressed, caused more extensive alterations in the Ab-V5 transcriptome than 0.05 mM MBOA, rendering 109 Differentially Expressed Genes (DEGs), including 80 DEGs in common (Fig. 1a). The 109 DEGs from 0.05 mM MBOA counted 19 upregulated and 90 downregulated DEGs, while 0.50 mM MBOA consisted of 105 upregulated and 180 downregulated DEGs (Fig. 1b). Despite deploying several annotation strategies, 109 out of 314 unique genes could not be identified (Table S2). Because of the scarcity of bacterial transcriptomics studies involving MBOA treatment, part of the genes that were not annotated are likely related to this condition.

**Fig 2:**
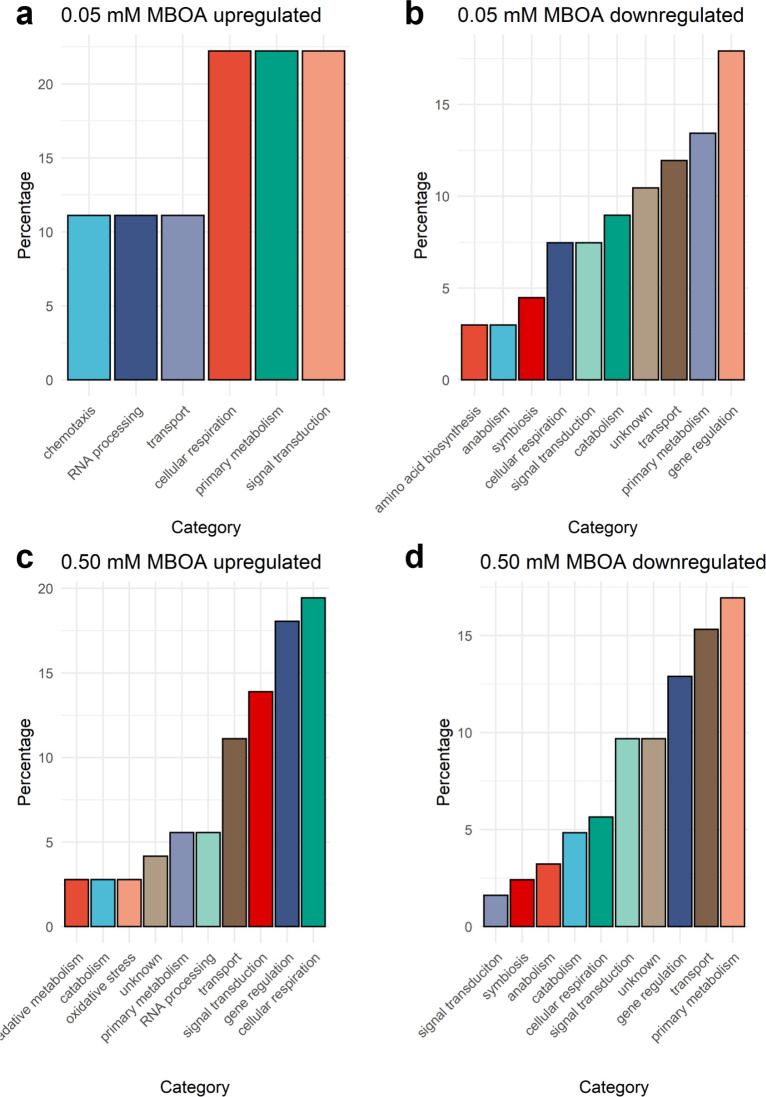
Principal functional classes of Differentially Expressed Genes (DEGs). (a, c) Upregulated DEGs; (b, d) downregulated DEGs identified in *Azospirillum brasilense* strain Ab-V5, according to the protein category. The percentage indicates the proportion of total annotated DEGs within each treatment (0.05 or 0.50). Blast2GO suite (55) was used to categorize the annotated genes via DIAMOND v2.1.7 (54) into functional Gene Ontology (GO) terms.

Upon exposure to MBOA, the transcriptomic profile of Ab-V5 exhibited pronounced alterations primarily in the domains of gene regulation, transport, primary metabolism, and signal transduction, sequentially (Fig. 2) (Fig, S4). This impact suggests that MBOA is perceived by Ab-V5, substantiated by the noteworthy number of DEGs identified under the categories of both signal transduction and gene regulation, thereby implying a potential role for MBOA as a signaling molecule. A complete list of the DEGs from the Ab-V5 RNAseq is available in Supplementary Data (Table S4).

### In Ab-V5, most upregulated genes are related to gene regulation and metabolic processes, while DEGs related to plant-microbe interactions are mainly downregulated

**Fig 3:**
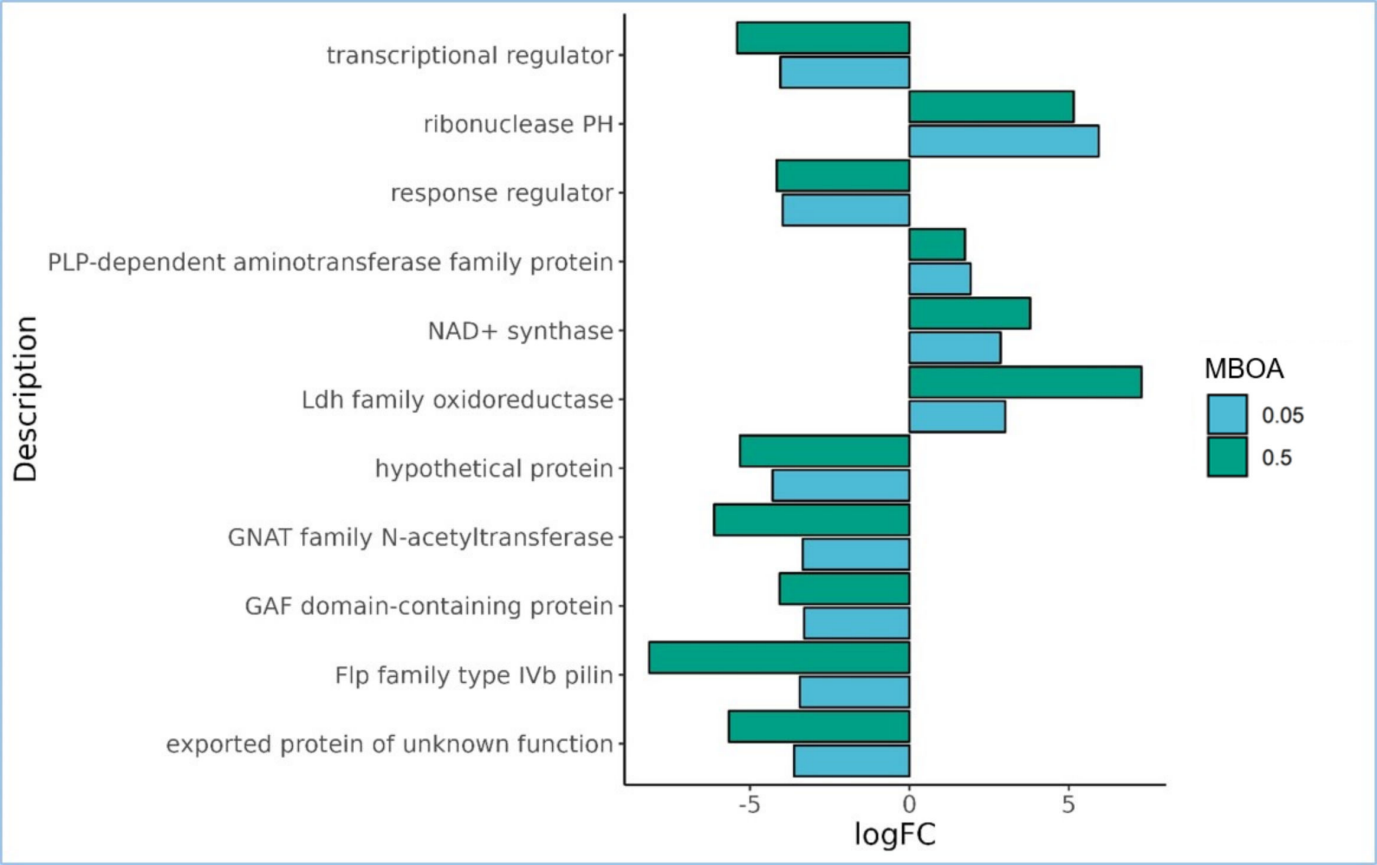
Common DEGs (*P* = 0.05) in treatments 0.05 and 0.50 of *Azospirillum brasilense* Ab-V5. DEGs displayed in the bar plot exhibit either positive or negative correlations with MBOA concentration. Downregulated DEGs were selected with a cut-off logFC value of −3 while no threshold was applied on common upregulated DEGs. The *x*-axis gives the expression values per gene for each gene with a description displayed on the *y*-axis. Blast2GO suite (55) was used to categorize the annotated genes via DIAMOND v2.1.7 (54) into functional Gene Ontology (GO) terms.

In concert with significant changes in gene expression within the primary metabolism category, it is noteworthy that the majority of the relatively upregulated DEGs are associated with gene regulation and cellular respiration (Fig. 2a and c). Most notable DEGs displaying the highest logFC values within this category are “Ldh family oxidoreductas” (AHNNBFGK_03305); “SDR family oxidoreductase” (AHNNBFGK_02025) and “NAD + synthase” (AHNNBFGK_00885) along the other 11 upregulated cellular respiration classified DEGs. The direct positive relationship observed between gene expression levels and MBOA concentration of AHNNBFGK_03305 and AHNNBFGK_00885 categorized under cellular respiration underscores the activation of energy metabolism within the cell (Fig. 3) (Fig. S3a and b).

**Fig 4:**
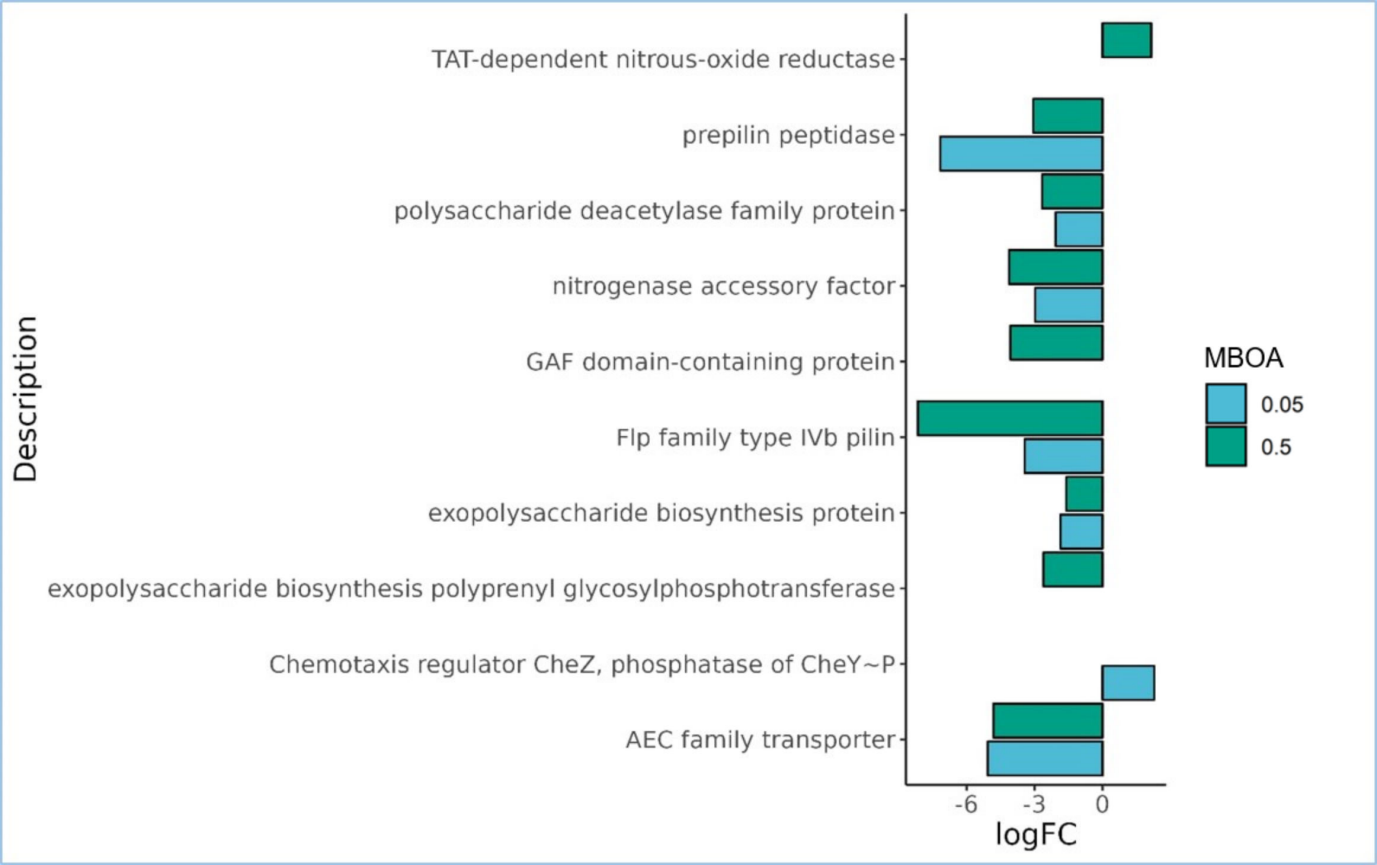
Plant-microbe interaction- related differentially expressed genes (DEGs) (*P* = 0.05) identified in *Azospirillum brasilense* Ab-V5, with logFC values for the treatments 0.05 mM and 0.50 mM MBOA. The *x*-axis gives the expression values in logFC per gene for each gene with description displayed on the *y*-axis. Blast2GO suite (55) was used to categorize the annotated genes via DIAMOND v2.1.7 (54) into functional Gene Ontology (GO) terms.

In our investigation, we grouped DEGs associated with plant-microbe interactions, recognizing the complexity of plant-microbe interactions as a phenomenon governed by intricate interplays. Therefore, we considered DEGs previously categorized under extracellular polymeric substance (EPS) biosynthesis, nitrogen metabolism, auxin homeostasis, and chemotaxis (Fig. 4) (Fig. S3c and d). The chemotaxis regulator CheZ (AHNNBFGK_04641), which exhibited a logFC of 2.29, is a specific phosphatase for CheY-P and plays a pivotal role in modulating the flagellar motor complex. Interestingly, our study reveals that the expression of nitrogen accessory proteins (AHNNBFGK_00521) is increasingly suppressed with a rise in MBOA concentration from 0.05 to 0.50 mM, with logFC values of −2.98 and −4.13, respectively (Fig. 4). Notably, only in the 0.50 mM treatment, the gene expression of “TAT-dependent nitrous-oxide reductase” (AHNNBFGK_05842) exhibits a logFC of 2.15 (Fig. 4). This enzyme catalyzes the final step in denitrification, reducing nitrous oxide (N_2_O) to dinitrogen (N_2_). Furthermore, we found that both the 0.05 and 0.50 mM MBOA treatments significantly repressed the gene expression of the “auxin efflux carrier protein” (AHNNBFGK_02785), with logFC values of −5.07 and −4.82, respectively (Fig. 4).

It’s noteworthy that the majority of the DEGs associated with plant-microbe interactions displayed downregulation. This includes the “Flp family type IV pilin” (AHNNBFGK_03152) and the enzyme responsible for processing precursor subunits for pilin assembly, known as “prepilin peptidase” (AHNNBFGK_03151) (64) with logFC values of −3.44 and −7.17 in 0.05 mM and the logFC values −8.16 and −3.06 in the 0.50 mM treatment, respectively (Fig. 4).

### Biofilm in Ab-V5 is transcriptionally regulated by MBOA according to a non-linear dose response

In both the 0.05 mM and 0.50 mM treatment, an EPS biosynthesis protein transcript (AHNNBFGK_05273) exhibited a slight relative downregulation with a logFC of −1.86 and −1.60, respectively (Fig. 4), while in the 0.50 mM MBOA treatment, an additional EPS biosynthesis protein transcript (AHNNBFGK_05278) with a logFC value of −2.61 was differentially expressed (Fig. 4). In summary, MBOA treatment does not directly promote plant-microbe interaction-related mechanisms other than chemotaxis based on the annotated DEGs. Noteworthy, other less characterized or unknown mechanisms related to plant-microbe interactions may yet be affected by genes that were not annotated.

**Fig 5:**
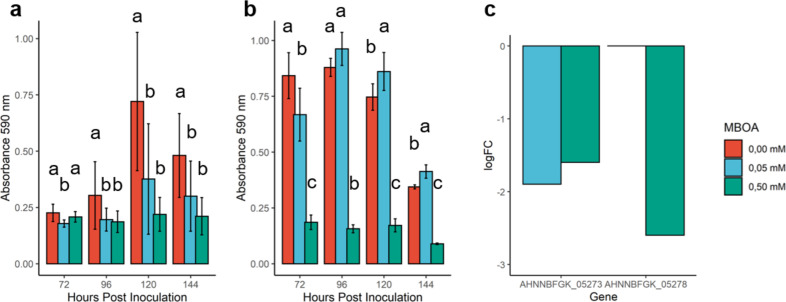
Biofilm formation in microtiter plates and gene expression levels of biofilm biosynthesis genes. (a) Biofilm production of *Pseudomonas protengens* Pf-5 and (b) *Azospirillum brasilense* Ab-V5 biofilm between 72 and 144 h post inoculation (hpi). Error bars in the charts represent standard deviation, different letters indicate significance at the level of 0.05 within each time point, calculated by a Kruskal-Wallis rank sum test. (c) Relative expression levels (logFC) of the genes AHNNBFGK_05273 (exopolysaccharide biosynthesis protein) and AHNNBFGK_05278 (exopolysaccharide biosynthesis polyprenyl glycosylphosphotransferase) calculated from 0.05 mM and 0.50 mM treated Ab-V5 RNA extracts after 72 hpi.

The relative downregulation of EPS biosynthesis-related DEGs, that contribute to biofilm formation, was confirmed by *in vitro* biofilm assays, demonstrating a negative influence of MBOA treatment on biofilm formation in both Ab-V5 and Pf-5 (Fig. 5). Ab-V5 samples for RNAseq were collected after 72 h of inoculation, but the physiological effect of the gene transcripts may require some time to be established. Therefore, we started measuring biofilm at the same timepoint as the samples for RNAseq were collected and measured biofilm with intervals of 24 h. In other studies, *A. brasilense* biofilm reaches the highest amount of biofilm after 96–120 h, corresponding to the time it takes to develop a mature biofilm, and diminishes slowly in the days after (65, 66); at 72 hpi, the biofilm formation, measured by *in vitro* assays, decreased as MBOA concentration increased, corresponding to the amount of downregulated EPS biosynthesis genes observed in the RNAseq data (Fig. 4). Additionally, the *in vitro* assays showed that the maximum amount of biofilm produced by Ab-V5 did not coincide among treatments, as it did with Pf-5 (Fig. 5A). In Ab-V5, the maximum amount of biofilm production was shifted 72 hpi from the control treatment to the 0.05 mM treatment after 120 hpi (Fig. 5B). Contrarily, strain Pf-5, which was less affected by MBOA (Fig. S1), displayed a strong decline in biofilm accumulation when treated with MBOA, while no DEGs related to biofilm were identified (Table S2).

### Ab-V5 is attracted to MBOA

**Fig 6:**
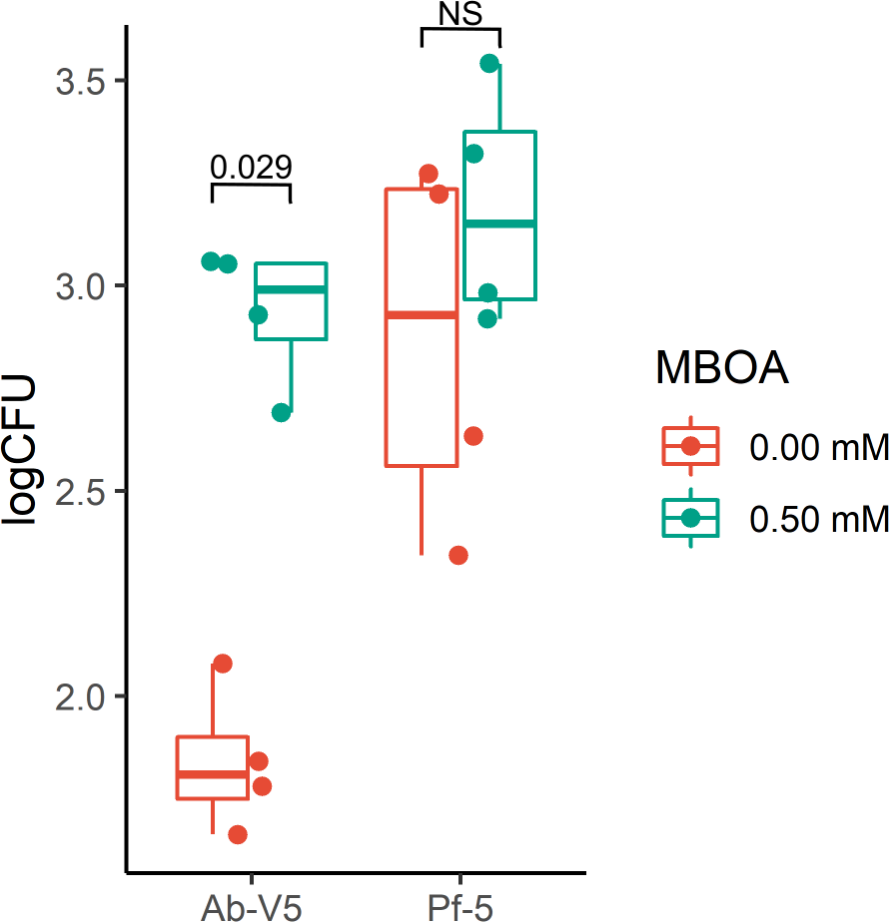
Chemotaxis assay with *Azospirillum brasilense* Ab-V5 and *Pseudomonas protegens* Pf-5 with 0.50 mM MBOA. Ab-V5 pre-cultures were washed and diluted in PBS to a final OD_600_ of 0.05 and used in a modified capillary assay. After 15 min of incubation at room temperature, the collected bacteria in 0.5 mL syringes were plated out and counted. *P*-values in the graph were calculated by a Wilcoxon rank sum exact test. NS, not significant (*P* = 0.05).

To validate the RNAseq results regarding the relative upregulation of *cheZ*, we performed a modified capillary assay for chemotaxis. We used the MBOA concentration of 0.50 mM which had a significant and moderate effect on the OD of Ab-V5 and Pf-5 cultures, respectively, in MBOA growth curves recorded over 12 h (Fig. S1). Ab-V5 was the only strain that exhibited a chemotactic response by accumulating a significantly higher number of CFU collected in the assay compared to the control (Fig. 6). Next, we analyzed chemotaxis in intermediate (0.05 mM) and high (0.50 mM) levels of MBOA using the same experimental setup, only with Ab-V5. Again, we observed a significant chemotactic response of Ab-V5, independent of the MBOA concentration used in the assay (Fig. S5).

**Fig 7:**
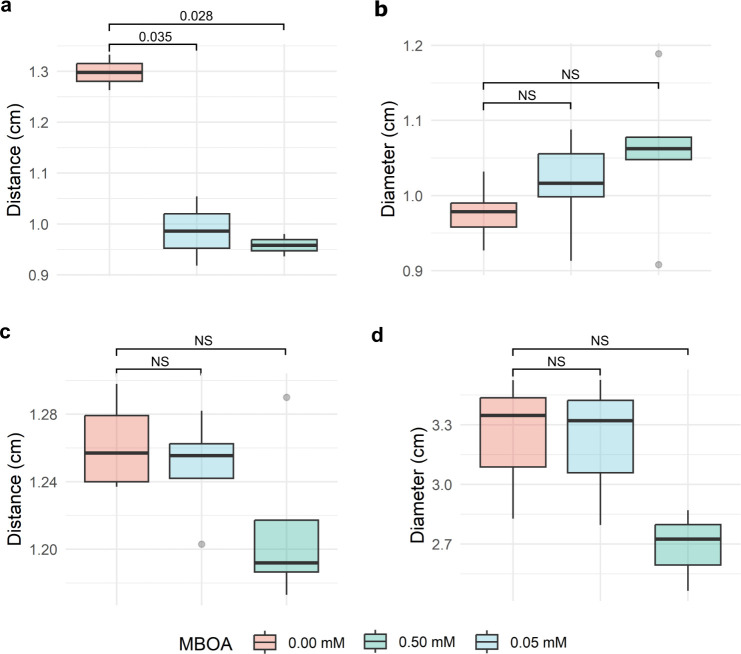
Swim plate and gradient plate assays with *A. brasilense* Ab-V5 and *P. protegens* Pf-5. **a** and **b** gradient plate and swim plate assays of Ab-V5, respectively. **c** and **d** gradient and swim plate assays of Pf-5. In the gradient plate assays (**a and c**), the distance of the bacterial culture to the agar plug is displayed in cm; in the swim plate assays (**b and d**), the diameter of the culture is given in cm. *P*-values indicated in the graph were calculated by ANOVA tests. NS, not significant (*P* = 0.05).

To test whether the positive result in the capillary assay was not caused by an increased motility of the bacteria, additional swim plate and gradient plate assays were carried out with Ab-V5 and Pf-5 on semi-solid minimal medium. With Pf-5, in both assays, no significant differences were detected (*P* = 0.05). In the case of Ab-V5, the swim plate assays did not indicate any differences among MBOA and control treatments (*P* = 0.05). However, the gradient assay showed that the distance was smaller between the Ab-V5 cultures and the 0.05 mM agar plug than the distance to the 0.00 mM MBOA containing agar plugs (*P* = 0.05) (Fig. 7). We can, thus, conclude that Ab-V5 is attracted to 0.05 mM MBOA while Pf-5 is not, and that the results observed from the capillary assay are likely not caused by improved motility, based on the results from the swim plate assays, but by chemoattraction.

### MBOA treatment improves biofilm formation by Ab-V5 on *Arabidopsis thaliana* roots

**Fig 8:**
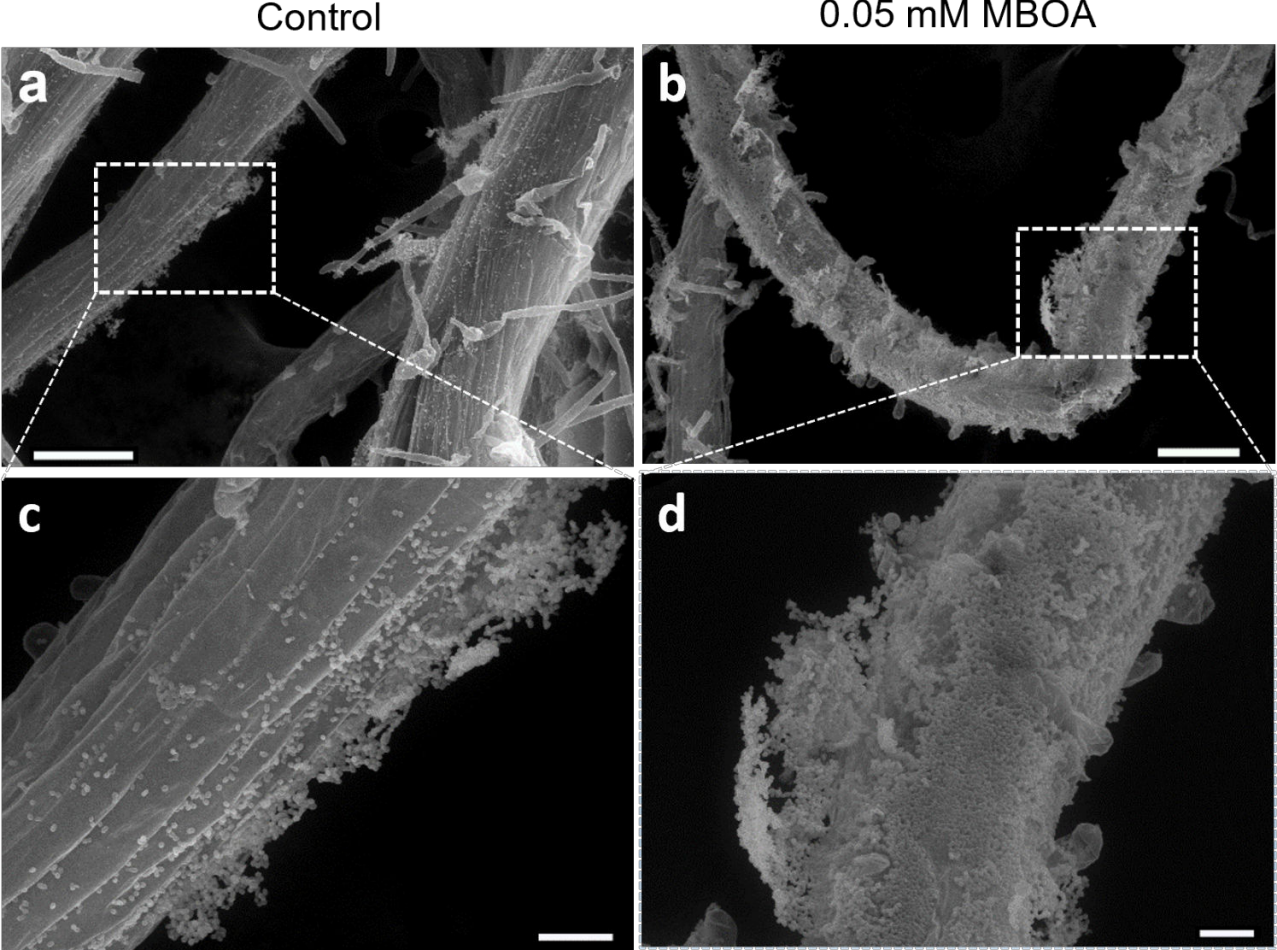
Scanning electron microscopy of *Arabidopsis thaliana* Col-0 roots inoculated with *Azospirillum brasilense* Ab-V5. Seedlings were grown on ½ MS agar medium for 14 days and incubated for 96 h with Ab-V5 cultures of OD_600_ 0.05, prior to sample preparation. (a and c) Control treatment of *Arabidopsis thaliana* Col-0 roots containing 0.5% acetone as a substitute for MBOA. (b and **d)** 0.05 mM MBOA treated *Arabidopsis thaliana* Col-0 roots. Scale bars indicate 100 µm (**a and b**) or 20 µm (**c and d**).

To complement the transcriptomics and biofilm data, we carried out live-cell imaging via epifluorescence microscopy and by scanning electron microscopy (SEM) on *A. thaliana* roots inoculated for 96 h with Ab-V5 and Pf-5. We observed that root samples treated with 0.05 mM MBOA showed a thicker and denser biofilm in Ab-V5, resulting in a greater coverage of the root surface than in the control treatment. Substantial amounts of biofilm were found in untreated samples, though not to the same extent as in the MBOA-treated roots (Fig. 8) (Fig. S6). In those samples, the surface area of biofilm-covered roots was approximately double that of the untreated roots. Pf-5 inoculated roots did not exhibit significant differences in the amount of bacterial biofilm on the root surfaces among treatments (Fig. S6). Both Ab-V5 and Pf-5 demonstrated ample colonization of root hairs and crevices in the root surface, independent of the MBOA treatment (Fig. S7).

### Peroxidase activity is unaffected by MBOA treatment

**Fig 9:**
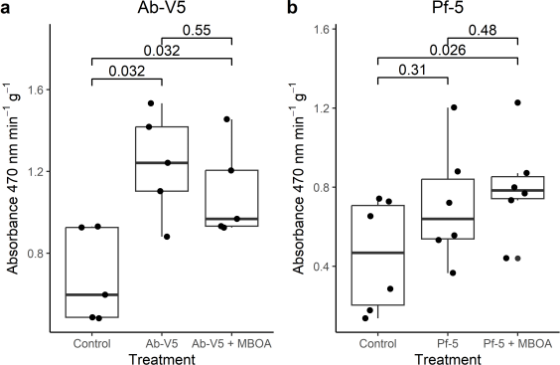
Peroxidase activity of *Arabidopsis thaliana* roots is enhanced when plants were inoculated with *Azospirillum brasilense* Ab-V5 (**a**) and with *Pseudomonas protegens* Pf-5 (**b**). Roots of *Arabidopsis* seedlings inoculated with either Ab-V5 or Pf-5 for 72 h were weighed, ground, and used for protein extraction. *P*-values indicated in the graph were calculated by ANOVA tests.

Colonization of *A. thaliana* roots by Ab-V5 caused an increase of one and a half times in the activity of peroxidases, both with and without the MBOA treatment. Thus, despite the effect of Ab-V5 inoculation, MBOA treatment did not influence *A. thaliana* peroxidase activity (Fig. 9a). Inoculation with Pf-5 resulted in a significantly elevated peroxidase activity compared to the sterile seedlings, albeit not as pronounced as the increase achieved with Ab-V5 inoculation. Interestingly, this augmentation was strictly observed in MBOA- treated *A. thaliana* as opposed to Pf-5 without MBOA, which did not show increased peroxidase activity compared to control (Fig. 9b).

## DISCUSSION 

### MBOA triggers a chemotactic response in Ab-V5

Given the overwhelming number of metabolites secreted from plant roots into the soil, recruitment of specific microorganisms is not straightforward. Attraction of bacteria to primary metabolites such as sugars and amino acids is short-lived and unspecific because they can be metabolized by a broad range of microorganisms (67, 68). MBOA, however, is a relatively stable compound (69) with a sustained release in the soil (5, 7). Intriguingly, exclusively in the lower concentration (0.05 mM) evaluated, the chemotaxis regulatory gene *cheZ* was relatively upregulated in Ab-V5 (Fig. 4), a result also obtained from the gradient plate assay while the *in vitro* chemotaxis assay showed that both 0.05 and 0.50 mM MBOA inflicted chemoattraction of Ab-V5 (Fig. 6 and Fig. 7). This possibly indicates a concentration-dependent signaling mechanism regulating chemotaxis. Since Ab-V5 will first encounter lower concentrations when moving along an MBOA gradient towards the root, chemotaxis-related proteins have been expressed, and the chemotaxis machinery will be established before reaching higher concentrations such as 0.50 mM, abolishing the need for further upregulation of chemotaxis genes. Furthermore, chemotaxis responses by the 0.50 mM dose can be regulated by different regulatory mechanisms than the 0,05 mM dose, such as by post-translational regulation of protein activity (70).

The *cheZ* gene product, CheZ, modulates the flagellar motor complex by dephosphorylation and inactivation of CheY which in its turn interacts with the switch domain of the flagellar motor, reverting the rotational direction of the flagellum (71, 72). Hence, Ab-V5 seems to be more sensitive to lower concentrations of MBOA, which possibly enables the strain to be attracted over longer distances. This enhanced sensitivity of MBOA in bacteria enables plants to reach more potential beneficial PGPB, since the number of bacteria in the soil multiplies by the third power with distance from the source, considering the soil environment as a homogeneous three-dimensional space.

### MBOA acts as a signaling molecule and stimulates energy metabolism

Our findings suggest that MBOA acts as a signaling molecule in Ab-V5 and causes substantial genetic reprogramming when exposed to MBOA-enriched environments (Fig. 2), with physiological implications that were evident in biofilm assessments and chemotaxis experiments. In contrast, alterations of the Pf-5 transcriptome were limited in the 0.50 mM MBOA treatment and undetected when subjected to the lowest concentration of MBOA (0.05 mM) (Table S2). These results of Pf-5 are in agreement with the responses to MBOA in the growth curves (Tables S1 and S2, Fig. S2), chemotaxis (Fig. 6 and 7), and biofilm assays (Fig. 5a).

The contrasting effects of the 0.05 mM and 0.50 mM MBOA treatments on Ab-V5 are evident in their impact on cellular respiration and energy metabolism. The 0.50 mM treatment notably stimulated cellular respiration, through glycolysis and by promoting oxidoreductases involved in the electron transport chain, substantiated by the positive correlation between the MBOA concentration and expression values of an “Ldh family oxidoreductase” gene (AHNNBFGK_03305) and an “NAD + synthase” gene (AHNNBFGK_00885) (Fig. 3). AHNNBFGK_03305 is an L-lactate dehydrogenase which is an enzyme known for its role in glycolysis, where it converts pyruvate to L-lactate, while AHNNBFGK_00885 is involved in NAD+ biosynthesis (73, 74). NAD + is known to play crucial roles in mediating redox reactions, electron transport, and as a substrate for poly-ADP-ribose polymerases (75). The absence of upregulated chemotaxis genes in the 0.50 mM treatment and results from the swim plate assays (Fig. 7) suggest that the increased energy generated through cellular respiration is likely not allocated to bacterial locomotion. Thus, investigating bioenergetics in this context would be an interesting avenue for future research.

### Mechanisms involving plant-microbe interactions are downregulated

We found that properties related to plant-microbe interactions were relatively inactive under MBOA regime (Fig. 4). Notably, biofilm biosynthesis-related genes were negatively correlated with MBOA concentration, and auxin release was diminished by relative downregulation of auxin efflux carriers. Therefore, the results suggest that the export of auxins produced by Ab-V5, which has a pivotal role in plant growth-promotion (76), is reduced under these conditions when compared to the control treatment.

Another hallmark of diazotrophic bacteria in plant-microbial interactions, is nitrogen fixation. *A. brasilense* is able to fix atmospheric nitrogen in the form of ammonium under micro-aerobic conditions. We found that in 0.50 mM MBOA, TAT-dependent nitrous-oxide reductase (AHNNBFGK_05842) was relatively upregulated, enhancing the conversion of nitrous-oxide to dinitrogen (N_2_). At the same time, a nitrogenase accessory factor (AHNNBFGK_00521) was relatively downregulated in both 0.05 and 0.50 mM MBOA, diminishing the reduction of N_2_ to ammonium (Fig. 4). Consequently, in 0.50 mM MBOA, this may lead to a local buildup of N_2_. N_2_, however, is unreactive and safe for the cell to store in large amounts (77) and, typically in the form of ammonium, is often a limiting nutrient for plant growth (78).

To exert growth promoting properties, many PGPB effectively require colonization of the roots from the interacting plant. By differences in root exudation patterns according to root zones and because of distinct chemotaxis and quorum sensing responses of bacteria, roots are occupied in a non-uniform distribution (79–82). Ab-V5 and Pf-5 showed preference for root hairs and crevices as primary colonization sites (Fig. S7). The colonization pattern of Pf-5 is comparable with *P. fluorescens* WCS365 which forms a thin biofilm localized around fissures, while *P. putida* produces a thick continuous biofilm spreading over the entire root (81, 83). After colonizing the root surface, endophytic bacteria internalize the plant tissue granting the advantage of a steady supply of nutrients in a protected environment. Penetration, however, does not necessarily require active mechanisms (84), but involves a range of bacterial traits (85), and may occur passively via entering through cracks and sites of lateral root emergence (86). Interestingly, once root colonization has been initiated, *A. brasilense* and *P. fluorescens* establish a positive feedback loop by stimulating BX metabolism of the plant (41, 42). Colonization by *A. brasilense* renders a species-specific readout of BX derivatives (41, 87), while inoculation of maize plants with *P. fluorescens* MZ05 causes induction of *BX2* and *GLU2,* two genes related to BX metabolism, augmenting BX content in leaves (42).

Our transcriptomic results suggest that plant MBOA exudation may stimulate recruitment more clearly than promoting direct root colonization mechanisms such as biofilm formation.

We observed that the MBOA treatment imposed a delay in biofilm formation by *in vitro* crystal violet assays (Fig. 5b), demonstrating the maximum amount of biofilm after 120 h of inoculation when treated with 0.05 mM, while after 72 h, the control treatment caused more biofilm to form by Ab-V5.

This delay in biofilm measured by the absorbance of crystal violet is likely to stem from different bacterial population dynamics of Ab-V5 when grown in *in vitro* conditions without agitation and by the lack of nutrients after 72 h of growth. Likewise, a delay in population growth was observed in bacterial cultures grown in statical conditions over the time span of 72 h (Table S1) (Fig. S2). This corroborates with the negative correlation between MBOA concentration and the number of DEGs related to biofilm biosynthesis that were identified by RNAseq (Fig. 5) carried out on RNA isolated from statically grown cultures after 72 h, coinciding with the amount of biofilm measured by crystal violet staining after 72 h (Fig. 5b). Microscopy assays in our study revealed that more biofilm formed on 0.05 mM MBOA-treated roots when inoculated with Ab-V5. In contrast to *in vitro* bacterial growth, bacterial growth on the root surface can be sustained by release of primary metabolites by the root. Host factors can, therefore, explain why results from microscopy investigations did not exactly match with the *in vitro* experiments.

In contrast to Ab-V5, in Pf-5, we could not identify any DEGs related to biofilm synthesis and biofilm measured by *in vitro* assays showed a linear correlation with MBOA concentration. Hence, in this case, MBOA treatment might have affected surface properties or the extracellular matrix composition of the biofilm, thereby changing the adherence and aggregation. Alternatively, MBOA can have influenced proteins involved in biofilm formation by direct interaction or interfered with signaling molecules regulating biofilm without changing gene expression. Hence, this all lead us to conclude that possibly, during early root colonization, Ab-V5 biofilm production is suppressed by MBOA on a transcriptional level. Furthermore, two DEGs related to pilin biosynthesis (AHNNBFGK_03151 and AHNNBFGK_03152) which are proteinaceous, polymeric appendages distinct from flagella, involved in the first steps of bacteria-host interactions (88), were in both conditions severely downregulated (Fig. 4). Hence, both transient absorption and permanent anchoring of the Ab-V5 were relatively downregulated by the MBOA treatment.

Concurrently, transcriptomics data of Ab-V5 showed that the highest number of upregulated DEGs in 0.50 mM MBOA was associated with cellular respiration (Fig. 2c). This underpins that MBOA treatment stimulates the motile bacterial lifestyle by diminishing the growth rate yet increasing cellular respiration. Thus, we surmise that energy spent on growth and duplication events is possibly allocated instead to chemotaxis and metabolic rewiring, depending on the MBOA concentration that was applied.

After 72 hpi with Ab-V5 and Pf-5, *A. thaliana* seedlings showed elevated peroxidase activity (Fig. 9). Since peroxidases keep ROS levels in check and protect cellular homeostasis (89–91), results may be indicative of a plausible indirect defense mechanism against phytopathogens (86). Fukami et al. (23) showed that treatment of maize plants with *A. brasilense* Ab-V5 stimulated jasmonic acid (JA) and salicylic acid (SA) pathways, leading to the activation of induced systemic resistance (ISR) as well as the expression of defense-related genes (22, 23), while ISR by Pf-5 is independent of SA signaling (92) and marked by increased peroxidase activity (93, 94), corroborating our results. Nevertheless, this effect was independent of the MBOA treatment.

### Physiological responses to MBOA may be linked to the ecological function of the bacteria

We surmise that the negative effect on bacterial growth, biofilm, and some related features of Ab-V5 most likely stem from applying relatively high concentrations of MBOA rather than being a limiting factor for Ab-V5-plant interaction. BXs are produced by many plants of the Poacea family and by some other dicotyledonous plants (1, 95, 96), but not in *A. thaliana* (97), which was used for our microscopy study. The BX concentration can vary considerably among plant tissue and between species. The grains of wheat and rye can contain around 4.8 µg g^−1^ dry weight (DW) (= 0.029 mM) and 95 µg g^−1^ DW (= 0.575 mM), respectively (98). In rye, shoots can accumulate 1,900 µg g^−1^ DW (= 11.505 mM) (99), while maize shoots may contain several mg g^−1^ DW (= 6.055 mM) (100, 101). Inside maize roots, around 2 µg g^−1^ fresh weight (FW) (= 0.005 mM) of HDMBOA-glc and around 1 µg g^−1^ FW (= 0.003 mM) of DIMBOA-glc accumulate between 2 and 3 weeks after germination (5), which spontaneously degrade into MBOA in the soil environment. Compared to the above-mentioned quantities of total BX extracts which were calculated per gram of DW plant tissue, the latter are expressed in µg g^−1^ of FW which will naturally be much lower because of the water content of fresh tissue. In the soil environment of maize plants, Hu et al*.* (7) measured 10 µg MBOA per 300 mL of soil (= 0.0002 mM), during the first 7 weeks after germination (7). Nonetheless, *A. brasilense* is frequently associated with BX-producing grass species and hence may be expected to be tolerant to MBOA (80, 102–104). Interestingly, Ab-V5 at the same time displayed a chemotactic response, facilitating rhizospheric establishment. In contrast, Pf-5, which was isolated from the cotton rhizosphere, supposedly free of BX, was little affected in terms of *in vitro* growth, chemotaxis, biofilm production, and transcriptome. One way to interpret these results could be that the sensitivity of Ab-V5 to MBOA enables recruitment of these PGPR by the plant through MBOA production. While Ab-V5 might be recruited from the bulk soil, MBOA release by cereal roots does not aid in Pf-5 establishment in the rhizosphere. Thus, considering the frequent occurrence of Ab-V5 with BX producing cereals, Ab-V5 is likely better adapted to BX content in the soil environment and might exploit MBOA signaling as a cue for localizing cereal roots.

In addition, the ecological function of the two strains may explain their differences in adaptation to BX production of the host plant. Since Ab-V5 fulfills a role as a growth promoting strain by nitrogen fixation (14) and plant hormone production (11–13), this strain is likely to exhibit a more precise and regulated interaction with its host plant, which has a narrower host range compared to the diverse plant hosts colonized by Pf-5 (31–33). Pf-5 provides an indirect benefit for plants by its biocontrol function (26–30), which suppresses a broad spectrum of pathogens and hence many plants profit from rhizosphere colonization by this strain. Thus, the more specific interaction of Ab-V5 with cereals creates the necessity of identifying the right plant host in the environment by chemical sensing and chemoattraction, a role that may be provided by BXs from cereal roots, in contrast to Pf-5, that is able to colonize the roots of many different plant species.

### Conclusion

Our results clearly show how MBOA acts in the first stages of Ab-V5 and Pf-5 plant interactions, including signal transduction, chemotaxis, and metabolic adaptation. In higher concentrations of MBOA (0.50 mM) or hypothetically in closer proximity to the roots where MBOA emanates, *A. brasilense* Ab-V5 experiences a metabolic reprogramming and prepares for transitioning to a lifestyle in close interaction with the host plant. Considering untreated Ab-V5 as the reference physiological state, energy homeostasis is strongly upregulated, allowing for a reallocation of energy for altering transport and rerouting metabolic networks. Nitrogen fixation is suppressed, and the gene expression of efflux carriers responsible for the release of auxins is reduced in comparison to MBOA-free Ab-V5. The sensitivity of Ab-V5 to MBOA may allow it to serve as a cue for locating specific plant hosts, a trait lacking in Pf-5, which exhibits a broader host range.

Considering its ecological impact and growth-promoting properties, *A. brasilense* interaction can highly benefit BX-producing cereals (12, 22, 23, 105–108). Moreover, MBOA harbors a considerable potential to attract PGPB and stimulate rhizosphere colonization, a promising aspect which has largely remained unexplored. The present study sheds light on the dynamics and interplay of the intricate mechanisms at play governing plant-microbial interactions, which help to understand the molecular function of MBOA. Bearing in mind that MBOA has a strong impact on microorganisms, MBOA treatment can be a promising tool for improving the inoculation success rate of specific strains and enhance crop health in agricultural settings (44, 109–111). To reveal how exactly those goals can be achieved and to what biofertilizer PGPR this strategy can be applicable, progression was made by advancing fundamental research on bacterial physiology.

## Data Availability

The datasets generated and/or analyzed during the current study are available in the ENA repository (https://www.ebi.ac.uk/ena/browser/home) under accession number ERP162234.

## SUPPLEMENTAL MATERIAL

Supplemental material mbio.01414-25-s0001.docx Supplemental figures and tables.

ASM does not own the copyrights to Supplemental Material that may be linked to, or accessed through, an article. The authors have granted ASM a non-exclusive, world-wide license to publish the Supplemental Material files. Please contact the corresponding author directly for reuse.
