# Supplementary material for: Benzoxazinoids stimulate chemotaxis and act as a signaling molecule in Azospirillum brasilense Ab-V5, while showing minor effects on Pseudomonas protegens Pf-5
Source: mBio. 2025 Jul 31;16(9):e01414-25. doi: 10.1128/mbio.01414-25 (PMC12421955; doi:10.1128/mbio.01414-25)
Supplement: Supplemental material — Supplemental figures and tables. [file mbio.01414-25-s0001.docx]

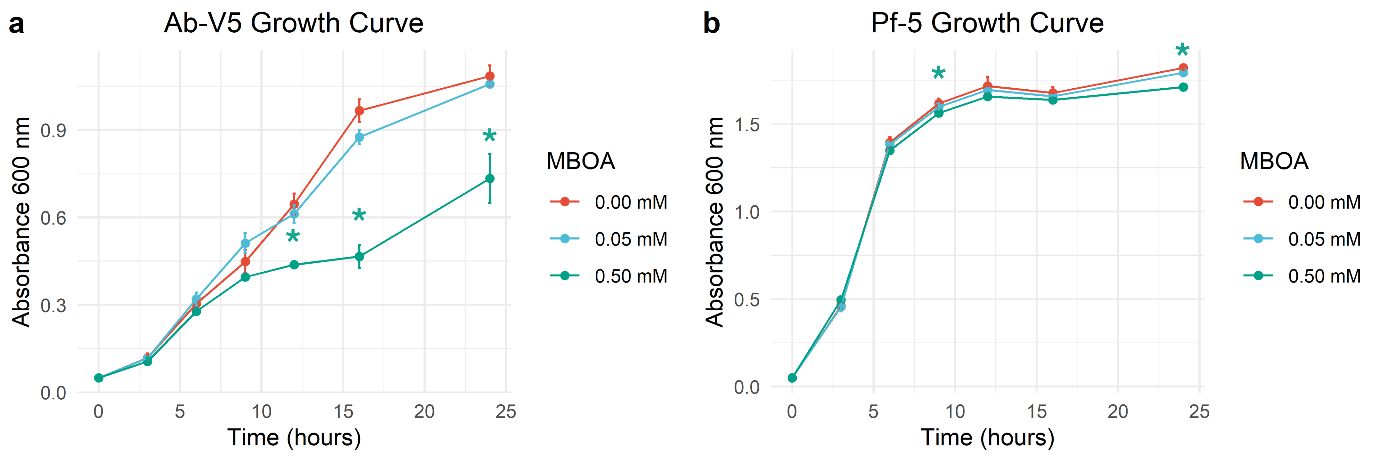


**Supplementary Figure 1:** Growth curves of PGPB strains **a** *A. brasilense* Ab-V5 and **b** *P. protegens* Pf-5 in a series of MBOA concentrations. Pre-cultures were diluted to a start OD of 0.05, and bacterial inoculums were supplemented with 20-fold concentrated MBOA suspension or an equal amount of acetone in control treatments. At an interval of three hours, at 16 h and 24 h, the OD was measured at 600 nm by spectrophotometry. Error bars in the graphs show standard deviation. Significant codes: p-values: ‘*’: 0,05.

**Supplementary Table 1**: Enumeration of colony forming units (CFU) by diluting and plating. *A. brasilense* Ab-V5 and *P. protegens* Pf-5 were grown statically amended with either 0.00 mM, 0.05 mM or 0.50 mM MBOA; incubated for 0, 24, 48 or 72 hours in triplicate and plated out on solid growth medium. Adjusted p-values comparing MBOA and control treatments were calculated by Kruskal-Wallis tests.

| **Strain** | **Time (h)** | | **MBOA (mM)** | | | **CFU (x 10⁸)** | | **SD** | **adjusted p-value** | |  |
| --- | --- | --- | --- | --- | --- | --- | --- | --- | --- | --- | --- |
| Ab-V5 | | 0 | | 0.00 | 12,33333333 | | 3,091206 | | |  | |
|  | |  | | 0.05 | 12,33333333 | | 3,091206 | | |  | |
|  | |  | | 0.50 | 12,33333333 | | 3,091206 | | |  | |
|  | | 24 | | 0.00 | 81 | | 29,06315 | | |  | |
|  | |  | | 0.05 | 56,66666667 | | 27,77689 | | | 1 | |
|  | |  | | 0.50 | 23,66666667 | | 9,533566 | | | 0,11 | |
|  | | 48 | | 0.00 | 80,33333333 | | 48,76018 | | |  | |
|  | |  | | 0.05 | 62,33333333 | | 37,50852 | | | 1 | |
|  | |  | | 0.50 | 23,33333333 | | 29,53341 | | | 0,41 | |
|  | | 72 | | 0.00 | 15,66666667 | | 13,69509 | | |  | |
|  | |  | | 0.05 | 26,66666667 | | 26,41128 | | | 1 | |
|  | |  | | 0.50 | 25,66666667 | | 27,01029 | | | 1 | |
| Pf-5 | | 0 | | 0.00 | 47,33333333 | | 27,52373 | | |  | |
|  | |  | | 0.05 | 47,33333333 | | 27,52373 | | |  | |
|  | |  | | 0.50 | 47,33333333 | | 27,52373 | | |  | |
|  | | 24 | | 0.00 | 97,33333333 | | 29,40899 | | |  | |
|  | |  | | 0.05 | 80,33333333 | | 15,96524 | | | 1 | |
|  | |  | | 0.50 | 166,3333333 | | 67,31188 | | | 1 | |
|  | | 48 | | 0.00 | 76,33333333 | | 18,62495 | | |  | |
|  | |  | | 0.05 | 60,33333333 | | 25,69479 | | | 1 | |
|  | |  | | 0.50 | 150,3333333 | | 14,5 | | | 1 | |
|  | | 72 | | 0.00 | 93 | | 26,1916 | | |  | |
|  | |  | | 0.05 | 105,3333333 | | 56,27512 | | | 1 | |
|  | |  | | 0.50 | 117,6666667 | | 9,285592 | | | 0,54 | |


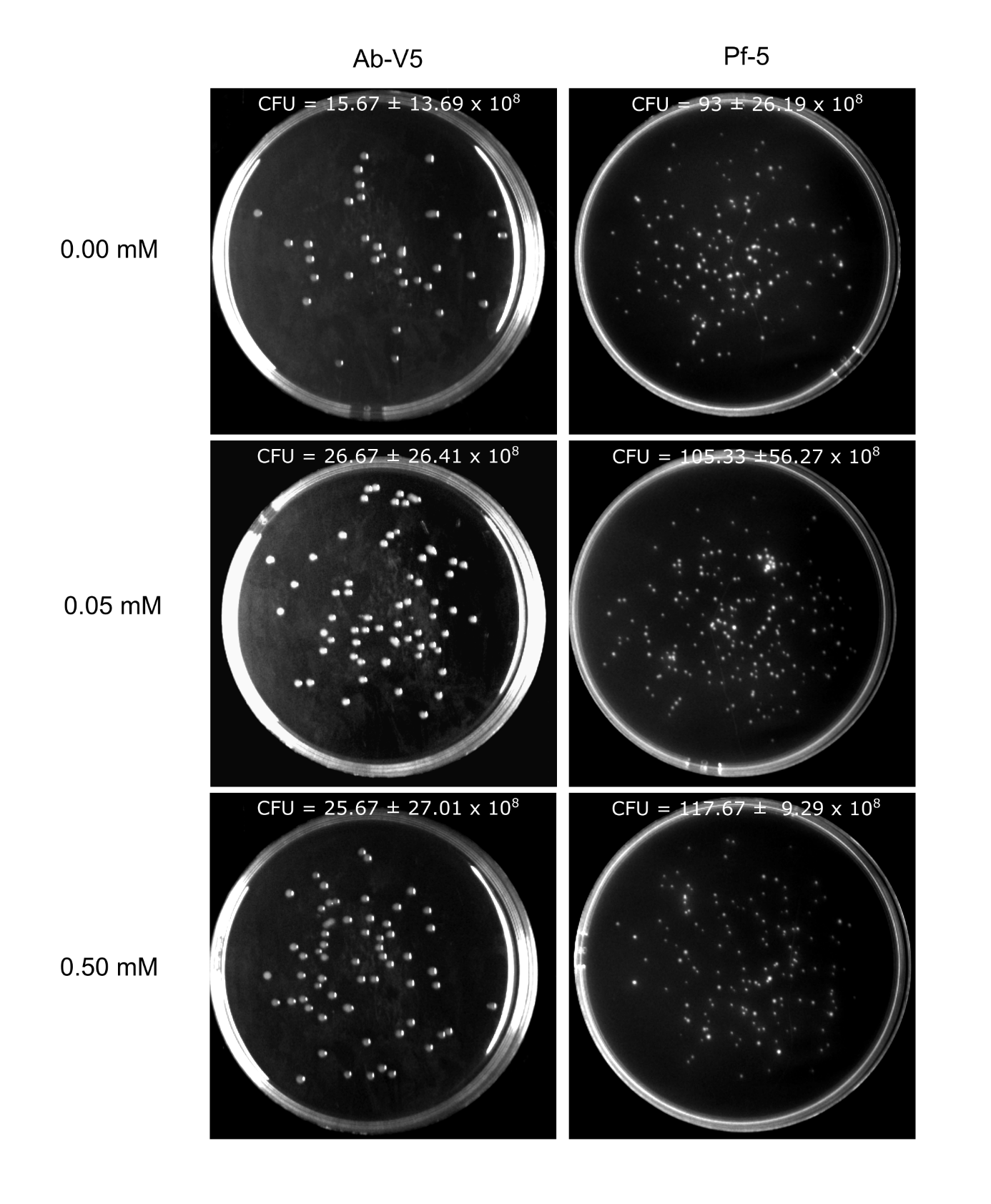


**Supplementary Figure 2**: Bacterial growth of *A. brasilense* Ab-V5 and *P. protegens* Pf-5 in statical conditions and treated with 0.00 mM MBOA and 0.5 % acetone, 0.05 mM or 0.50 mM MBOA. Plates of 10^8^-fold dilutions are shown after 72 hours of incubation the amount of colony forming units (CFU) indicated as the average of three repeats. Statistical analysis was carried out by a Kruskal-Wallis test comparing CFU among treatments at the specified timepoints, but no significant differences were found (p = 0.05).

**Supplementary Table 2:** Read library characteristics from the Illumina NextSeq sequencing. Per treatment, the three best samples were selected and sequenced. The average read count was calculated after trimming with Trimmomatic^49^ and filtering with RiboDetector^50^.

| PGPR | MBOA treatment | Average # reads | # DEGs | # Annotated DEGs | # Unidentified DEGs | % DEGs annotated |
| --- | --- | --- | --- | --- | --- | --- |
| Ab-V5 | 0.05 mM | 5025747 | 110 (81 shared) | 69 | 36 (23 shared) | 64 |
|  | 0.50 mM | 2947086 | 286 (81 shared) | 180 | 96 (23 shared) | 66 |
| Pf-5 | 0.05 mM | 11373198 | 0 | 0 | 0 | 0 |
|  | 0.50 mM | 10240785 | 8 | 6 | 2 | 75 |

**
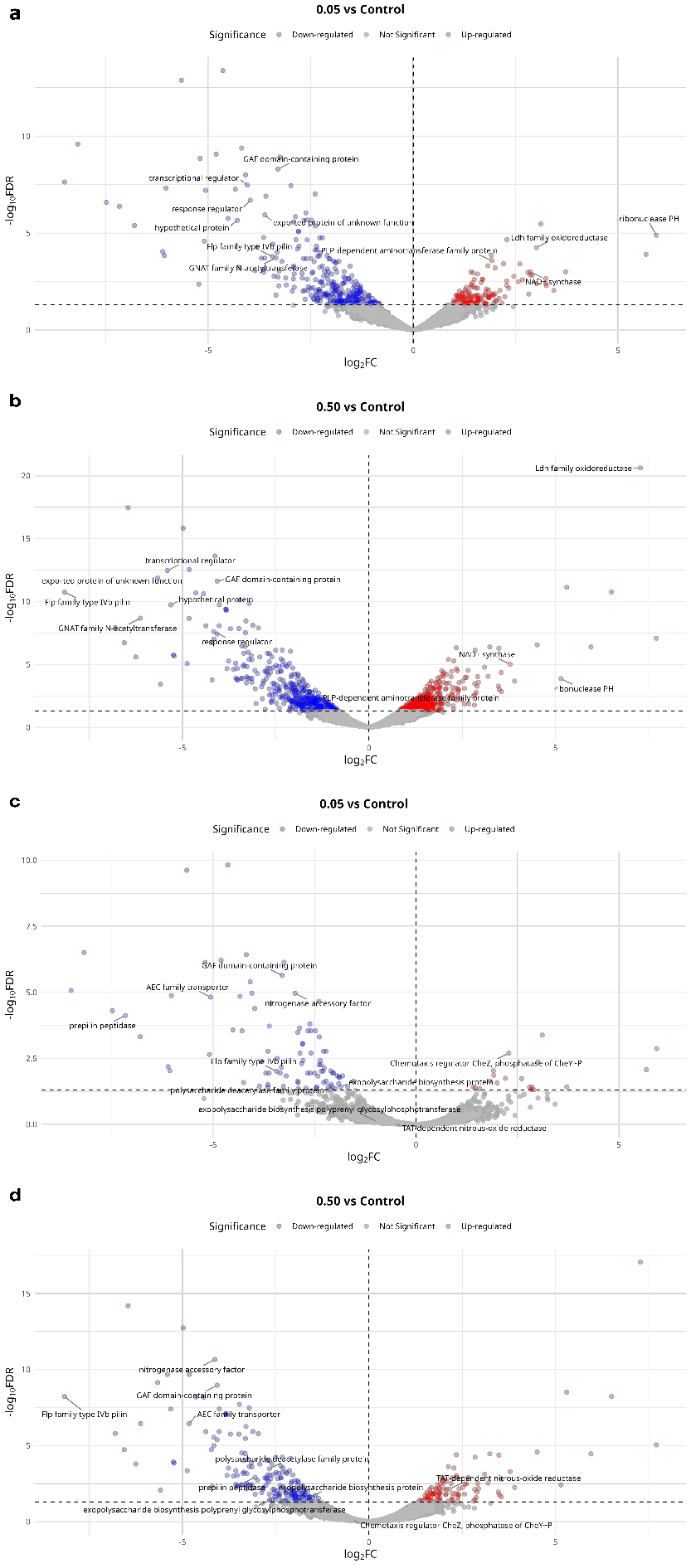
**

**Supplementary Figure 3:** Volcano plots of the relative expression values (-logFDR) compared to control treatments from RNAseq on *A. brasilense* Ab-V5. A threshold value of logFDR 2 was set for considering relevant differentially expressed genes (DEGs). **a** and **b:** DEGs that show a correlation between MBOA concentration and expression value. **c** and **d:** DEGs that are related to plant-microbial interactions.

**Supplementary Table 3:** Differentially Expressed Genes (DEGs) identified in the 0.50 mM MBOA treatment on *P. protegens* Pf-5 (p = 0.05). Blast2GO suite^55^ was used to categorize the annotated genes via DIAMOND v2.1.7^54^ into functional Gene Ontology (GO) terms.

| **Genes** | **Description** | **Category** | **logFC** |
| --- | --- | --- | --- |
| WP_011334291.1 | NAD(+) diphosphatase | cellular respiration | 1,16 |
| WP_016702562.1 | taurine dioxygenase | cellular respiration | -1,11 |
| WP_017336877.1 | L-threonine dehydrogenase | cellular respiration | 1,14 |
| WP_011058481.1 | inhibitor of vertebrate lysozyme family protein | housekeeping enzyme | 0,92 |
| WP_011059792.1 | sulfurtransferase TusA | sulfer metabolism | 1,38 |
| WP_011058772.1 | lipocalin family protein | Transport | 1,47 |
| WP_011059507.1 | DUF4197 domain-containing protein | Unknown | 1,22 |
| WP_011062008.1 | DUF2474 domain-containing protein | Unknown | 0,84 |


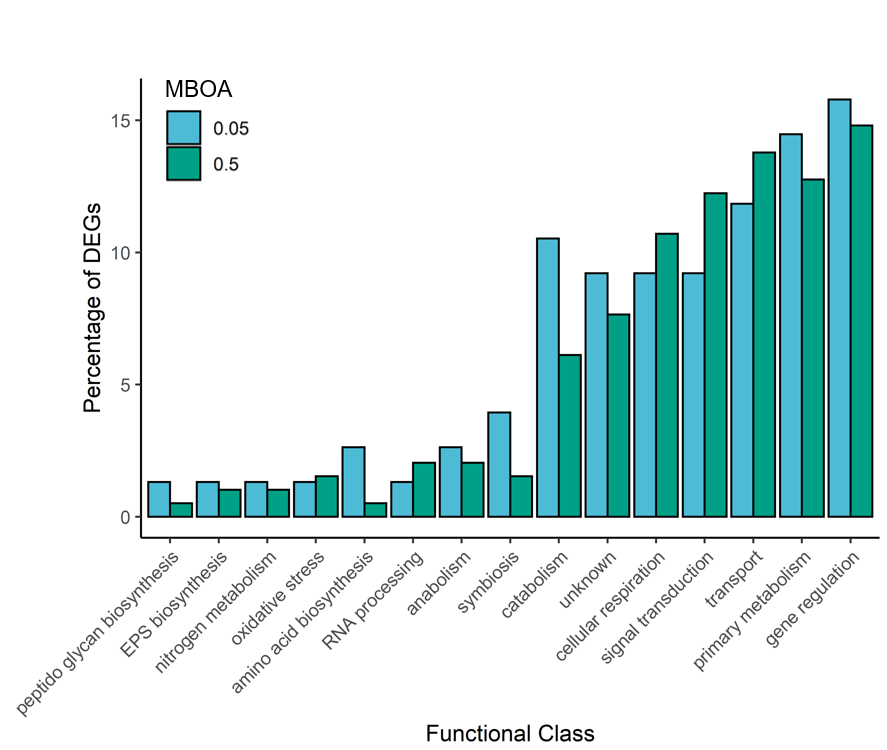


**Supplementary Figure 4:** Organization of all common differentially expressed genes (DEGs) found in 0.05 and 0.50 mM MBOA treatments of *Azospirillum brasilense* Ab-V5 relative to the control treatment. In each treatment DEGs are grouped per functional class displayed on the X axis and the Y axis gives the percentage of DEGs represented in each class. Blast2GO suite^55^ was used to categorize the annotated genes via DIAMOND v2.1.7^54^ into functional Gene Ontology (GO) terms. The unknown class contains DEGs with insufficient data to fit a functional class, but were annotated with unspecific information.

**Supplementary Table 4:** Overview of all Differentially Expressed Genes (DEGs) identified in both 0.05 mM and 0.50 mM MBOA treatments (p = 0.05). Blast2GO suite^55^ was used to categorize the annotated genes via DIAMOND v2.1.7^54^ into functional Gene Ontology (GO) terms.

| Genes | Description | Category | logFC 0,05 | logFC 0,50 |
| --- | --- | --- | --- | --- |
| AHNNBFGK_03470 | histidine biosynthesis protein | amino acid biosynthesis | -2,34 | -2,72 |
| AHNNBFGK_00590 | formyltetrahydrofolate deformylase | amino acid biosynthesis | -2,87 | -3,84 |
| AHNNBFGK_03476 | riboflavin biosynthesis protein RibD | anabolism | -2,80 | -4,01 |
| AHNNBFGK_00938 | D-glycero-beta-D-manno-heptose-7-phosphate kinase | anabolism | -4,34 | -3,71 |
| AHNNBFGK_02785 | AEC family transporter | auxin efflux carrier | -5,07 | -4,82 |
| AHNNBFGK_05533 | threonine/serine dehydratase | catabolism | -2,36 | -2,67 |
| AHNNBFGK_02037 | alpha/beta fold hydrolase | catabolism | -2,59 | -3,31 |
| AHNNBFGK_00345 | DUF1624 domain-containing protein | catabolism | -2,61 | -3,14 |
| AHNNBFGK_02106 | 2-hydroxychromene-2-carboxylate isomerase | catabolism | -3,81 | -2,52 |
| AHNNBFGK_04590 | DUF4743 domain-containing protein | catalytic domain | -2,29 | -2,07 |
| AHNNBFGK_00685 | ATP-binding protein | catalytic membrane protein | -5,20 | -4,64 |
| AHNNBFGK_03305 | Ldh family oxidoreductase | cellular respiration | 3,01 | 7,28 |
| AHNNBFGK_00885 | NAD+ synthase | cellular respiration | 2,86 | 3,78 |
| AHNNBFGK_01223 | SDR family oxidoreductase | cellular respiration | -1,54 | -2,10 |
| AHNNBFGK_05453 | NADH-quinone oxidoreductase subunit NuoF | cellular respiration | -1,99 | -1,96 |
| AHNNBFGK_06212 | alanine dehydrogenase | cellular respiration | -2,17 | -1,79 |
| AHNNBFGK_02723 | NAD(P)H-hydrate dehydratase | cellular respiration | -2,28 | -2,07 |
| AHNNBFGK_00074 | Ldh family oxidoreductase | cellular respiration | -2,50 | -2,39 |
| AHNNBFGK_05273 | exopolysaccharide biosynthesis protein | EPS biosynthesis | -1,86 | -1,60 |
| AHNNBFGK_04555 | RNA methyltransferase | gene regulation | -2,10 | -2,44 |
| AHNNBFGK_02618 | LysR family transcriptional regulator | gene regulation | -2,10 | -2,31 |
| AHNNBFGK_00239 | response regulator transcription factor | gene regulation | -2,28 | -1,99 |
| AHNNBFGK_04248 | IS5/IS1182 family transposase | gene regulation | -2,35 | -2,42 |
| AHNNBFGK_05050 | response regulator transcription factor | gene regulation | -2,62 | -3,47 |
| AHNNBFGK_04633 | response regulator transcription factor | gene regulation | -2,67 | -3,30 |
| AHNNBFGK_02659 | response regulator transcription factor | gene regulation | -2,68 | -2,30 |
| AHNNBFGK_03502 | GNAT family N-acetyltransferase | gene regulation | -3,35 | -6,13 |
| AHNNBFGK_03041 | transcriptional regulator | gene regulation | -4,05 | -5,41 |
| AHNNBFGK_03501 | GNAT family N-acetyltransferase | gene regulation | -4,09 | -3,82 |
| AHNNBFGK_00521 | nitrogenase accessory factor | nitrogen metabolism | -2,98 | -4,13 |
| AHNNBFGK_06193 | endolytic transglycosylase MltG | peptido glycan biosynthesis | -1,81 | -2,13 |
|  |  |  | Continuous | |

| Genes | Description | Category | logFC 0,05 | logFC 0,50 |
| --- | --- | --- | --- | --- |
| AHNNBFGK_01257 | PLP-dependent aminotransferase family protein | primary metabolism | 1,92 | 1,74 |
| AHNNBFGK_02823 | 2-keto-4-methylthiobutyrate aminotransferase | primary metabolism | -1,89 | -2,30 |
| AHNNBFGK_06222 | xanthine dehydrogenase family protein molybdopterin-binding subunit | primary metabolism | -2,03 | -1,60 |
| AHNNBFGK_06062 | 50S ribosomal protein L31 | primary metabolism | -2,46 | -3,27 |
| AHNNBFGK_01002 | aspartate aminotransferase family protein | primary metabolism | -2,48 | -2,50 |
| AHNNBFGK_03675 | glycoside hydrolase | primary metabolism | -2,80 | -2,36 |
| AHNNBFGK_01483 | carboxylating nicotinate-nucleotide diphosphorylase | primary metabolism | -2,91 | -3,28 |
| AHNNBFGK_05280 | Glycosyltransferase | primary metabolism | -3,71 | -3,14 |
| AHNNBFGK_02796 | Glycosyltransferase | primary metabolism | -7,49 | -3,57 |
| AHNNBFGK_04202 | ribonuclease PH | RNA processing | 5,93 | 5,15 |
| AHNNBFGK_03828 | serine/threonine-protein kinase | signal transduction | -2,22 | -2,21 |
| AHNNBFGK_00686 | HAMP domain-containing protein | signal transduction | -2,92 | -2,54 |
| AHNNBFGK_03599 | GAF domain-containing protein | signal transduction | -3,30 | -4,07 |
| AHNNBFGK_00240 | response regulator | signal transduction | -3,98 | -4,16 |
| AHNNBFGK_05283 | polysaccharide deacetylase family protein | symbiosis | -2,07 | -2,67 |
| AHNNBFGK_03152 | Flp family type IVb pilin | symbiosis | -3,44 | -8,16 |
| AHNNBFGK_03151 | prepilin peptidase | symbiosis | -7,17 | -3,06 |
| AHNNBFGK_01446 | MFS transporter | transport | -2,59 | -3,11 |
| AHNNBFGK_00994 | DHA2 family efflux MFS transporter permease subunit | transport | -2,71 | -2,83 |
| AHNNBFGK_00964 | microcin ABC transporter ATP-binding protein | transport | -2,82 | -3,36 |
| AHNNBFGK_02170 | cobalt transporter | transport | -2,92 | -3,83 |
| AHNNBFGK_05150 | Na/Pi cotransporter family protein | transport | -3,26 | -3,21 |
| AHNNBFGK_02107 | potassium channel protein | transport | -4,81 | -4,37 |
| AHNNBFGK_01499 | tetratricopeptide repeat protein | unknown | -2,79 | -2,64 |
| AHNNBFGK_01737 | family Rossman fold protein | unknown | -3,20 | -2,54 |
| AHNNBFGK_03451 | hypothetical protein | unknown | -3,32 | -2,99 |
| AHNNBFGK_03043 | exported protein of unknown function | unknown | -3,62 | -5,67 |
| AHNNBFGK_00671 | hypothetical protein | unknown | -4,29 | -5,32 |
|  |  |  | Conitnuation | |

| Genes | Description | Category | logFC 0,05 | logFC 0,50 |
| --- | --- | --- | --- | --- |
| AHNNBFGK_05480 | hypothetical protein | unknown | -4,52 | -3,77 |
| AHNNBFGK_00758 | alpha/beta hydrolase | catabolism | -3,27 |  |
| AHNNBFGK_02937 | alpha/beta hydrolase | catabolism | -2,06 |  |
| AHNNBFGK_04641 | Chemotaxis regulator CheZ, phosphatase of CheY~P | chemotaxis | 2,29 |  |
| AHNNBFGK_01133 | DNA polymerase III subunit epsilon | DNA replication | -1,91 |  |
| AHNNBFGK_03107 | GNAT family N-acetyltransferase | gene regulation | -3,65 |  |
| AHNNBFGK_04632 | transcriptional activator, LuxR/FixJ family | gene regulation | -2,22 |  |
| AHNNBFGK_05389 | pyrroloquinoline quinone biosynthesis protein PqqE | oxidative stress | -2,90 |  |
| AHNNBFGK_00736 | glycosyltransferase family 1 protein | primary metabolism | -2,45 |  |
| AHNNBFGK_04979 | aspartate carbamoyltransferase catalytic subunit | primary metabolism | 2,00 |  |
| AHNNBFGK_03203 | histidine kinase (plasmid) | signal transduction | 1,41 |  |
| AHNNBFGK_04279 | DUF4880 domain-containing protein | signal transduction | 2,91 |  |
| AHNNBFGK_05265 | hybrid sensor histidine kinase/response regulator | signal transduction | -2,42 |  |
| AHNNBFGK_03149 | type II and III secretion system protein family protein | transport | -3,65 |  |
| AHNNBFGK_04404 | MFS transporter | transport | 2,60 |  |
| AHNNBFGK_05590 | ABC transporter ATP-binding protein | transport | -6,12 |  |
| AHNNBFGK_03549 | hypothetical protein | unknown | -6,07 |  |
| AHNNBFGK_04770 | alpha,alpha-trehalose-phosphate synthase (UDP-forming) | abiotic stress |  | -1,78 |
| AHNNBFGK_03471 | ATP-grasp domain-containing protein | anabolism |  | -5,24 |
| AHNNBFGK_05366 | Aminotransferase | anabolism |  | -1,86 |
| AHNNBFGK_00988 | glutathione S-transferase | biodegradative metabolism |  | -2,22 |
| AHNNBFGK_01001 | glutathione S-transferase | biodegradative metabolism |  | 1,54 |
| AHNNBFGK_05412 | glutathione S-transferase family protein | biodegradative metabolism |  | 1,91 |
| AHNNBFGK_05326 | glycerol dehydrogenase | carbon catabolsim |  | 2,08 |
| AHNNBFGK_02106 | 2-hydroxychromene-2-carboxylate isomerase | catabolism |  | -2,52 |
| AHNNBFGK_02998 | alpha-galactosidase | catabolism |  | -1,91 |
| AHNNBFGK_04102 | DUF1206 domain-containing protein | catabolism |  | 2,41 |
| AHNNBFGK_06116 | D-amino acid dehydrogenase | catabolism |  | -2,12 |
|  |  |  | Continuation | |
|  |  |  |  |  |

| Genes | Description | Category | logFC 0,05 | logFC 0,50 |
| --- | --- | --- | --- | --- |
| AHNNBFGK_00661 | DUF4347 domain-containing protein | catalytic domain |  | 2,94 |
| AHNNBFGK_00070 | acetoin dehydrogenase dihydrolipoyllysine-residue acetyltransferase subunit | cellular respiration |  | -2,67 |
| AHNNBFGK_00188 | cytochrome c oxidase subunit I | cellular respiration |  | -1,78 |
| AHNNBFGK_00361 | formate dehydrogenase subunit gamma | cellular respiration |  | 2,80 |
| AHNNBFGK_00725 | 4-hydroxybenzoate octaprenyltransferase | cellular respiration |  | 2,84 |
| AHNNBFGK_00799 | NAD(P)/FAD-dependent oxidoreductase | cellular respiration |  | 2,66 |
| AHNNBFGK_01240 | energy transducer TonB | cellular respiration |  | 1,38 |
| AHNNBFGK_01707 | succinate dehydrogenase flavoprotein subunit | cellular respiration |  | 1,79 |
| AHNNBFGK_02025 | SDR family oxidoreductase | cellular respiration |  | 4,51 |
| AHNNBFGK_02251 | NAD(P)/FAD-dependent oxidoreductase | cellular respiration |  | 1,76 |
| AHNNBFGK_02473 | SDR family oxidoreductase | cellular respiration |  | 1,67 |
| AHNNBFGK_02615 | Gfo/Idh/MocA family oxidoreductase | cellular respiration |  | -6,56 |
| AHNNBFGK_04526 | cytochrome c | cellular respiration |  | 2,20 |
| AHNNBFGK_04675 | NAD(P)-dependent oxidoreductase | cellular respiration |  | 2,12 |
| AHNNBFGK_05452 | NADH-quinone oxidoreductase subunit G | cellular respiration |  | 1,88 |
| AHNNBFGK_05920 | cytochrome c oxidase subunit II | cellular respiration |  | -1,57 |
| AHNNBFGK_01721 | ATP-dependent DNA helicase | DNA replication |  | 2,41 |
| AHNNBFGK_05278 | exopolysaccharide biosynthesis polyprenyl glycosylphosphotransferase | EPS biosynthesis |  | -2,61 |
| AHNNBFGK_01464 | 3-oxoacyl-ACP reductase | fatty acid biosynthesis |  | -2,46 |
| AHNNBFGK_02843 | GFA family protein | formaldehyde degradation |  | 2,07 |
| AHNNBFGK_00642 | LysR family transcriptional regulator | gene regulation |  | -1,76 |
| AHNNBFGK_01092 | pirin family protein | gene regulation |  | 1,99 |
| AHNNBFGK_01239 | response regulator transcription factor | gene regulation |  | 1,99 |
| AHNNBFGK_01719 | XRE family transcriptional regulator | gene regulation |  | 3,55 |
| AHNNBFGK_02366 | LysR family transcriptional regulator | gene regulation |  | 2,88 |
| AHNNBFGK_02566 | LysR family transcriptional regulator | gene regulation |  | -2,23 |
| AHNNBFGK_02799 | winged helix-turn-helix transcriptional regulator | gene regulation |  | 2,39 |
| AHNNBFGK_03042 | response regulator transcription factor | gene regulation |  | -2,28 |
|  |  |  | Continuation | |
|  |  |  |  |  |

| Genes | Description | Category | logFC 0,05 | logFC 0,50 |
| --- | --- | --- | --- | --- |
| AHNNBFGK_03410 | N-acetyltransferase | gene regulation |  | -2,34 |
| AHNNBFGK_04004 | class I SAM-dependent RNA methyltransferase | gene regulation |  | 1,83 |
| AHNNBFGK_04282 | IS6 family transposase | gene regulation |  | 3,47 |
| AHNNBFGK_05158 | transcriptional repressor | gene regulation |  | -6,25 |
| AHNNBFGK_05175 | Crp/Fnr family transcriptional regulator | gene regulation |  | 2,62 |
| AHNNBFGK_05510 | GntR family transcriptional regulator | gene regulation |  | 1,58 |
| AHNNBFGK_05822 | LysR family transcriptional regulator | gene regulation |  | -1,99 |
| AHNNBFGK_05828 | Crp/Fnr family transcriptional regulator | gene regulation |  | 5,95 |
| AHNNBFGK_05980 | GNAT family N-acetyltransferase | gene regulation |  | 1,67 |
| AHNNBFGK_05992 | GNAT family N-acetyltransferase | gene regulation |  | 1,84 |
| AHNNBFGK_01932 | ferritin-like domain-containing protein | iron metabolism |  | -2,66 |
| AHNNBFGK_01036 | membrane protein | membrane protein |  | 1,91 |
| AHNNBFGK_04629 | YccF domain-containing protein | membrane protein |  | -2,00 |
| AHNNBFGK_05026 | hydrogenase maturation nickel metallochaperone HypA | nickel homeostasis |  | -1,65 |
| AHNNBFGK_04727 | Peptidase | nitrogen metabolism |  | -2,60 |
| AHNNBFGK_05842 | TAT-dependent nitrous-oxide reductase | nitrogen metabolism |  | 2,15 |
| AHNNBFGK_00723 | glutamate--cysteine ligase | oxidative stress |  | 1,65 |
| AHNNBFGK_03666 | Rubrerythrin | oxidative stress |  | -3,35 |
| AHNNBFGK_05844 | rubrerythrin family protein | oxidative stress |  | 6,50 |
| AHNNBFGK_00030 | CCA tRNA nucleotidyltransferase | primary metabolism |  | 1,71 |
| AHNNBFGK_00830 | 16S rRNA (guanine(966)-N(2))-methyltransferase RsmD | primary metabolism |  | -2,09 |
| AHNNBFGK_00837 | Dihydroorotase | primary metabolism |  | -2,01 |
| AHNNBFGK_01485 | UDP-galactopyranose mutase | primary metabolism |  | -3,22 |
| AHNNBFGK_02249 | Glycosyltransferase | primary metabolism |  | -1,59 |
| AHNNBFGK_02791 | nucleotide sugar dehydrogenase | primary metabolism |  | -2,49 |
| AHNNBFGK_02803 | MBL fold metallo-hydrolase | primary metabolism |  | -2,09 |
| AHNNBFGK_02809 | sigma-70 family RNA polymerase sigma factor | primary metabolism |  | -2,03 |
| AHNNBFGK_02852 | isochorismatase family protein | primary metabolism |  | -2,51 |
|  |  |  | Continuation | |
|  |  |  |  |  |

| Genes | Description | Category | logFC 0,05 | logFC 0,50 |
| --- | --- | --- | --- | --- |
| AHNNBFGK_04706 | HPF/RaiA family ribosome-associated protein | primary metabolism |  | -1,74 |
| AHNNBFGK_04846 | glycosyltransferase family 61 protein | primary metabolism |  | 1,65 |
| AHNNBFGK_05149 | NAD-dependent epimerase/dehydratase family protein | primary metabolism |  | -2,36 |
| AHNNBFGK_05530 | HAD family hydrolase | primary metabolism |  | 5,30 |
| AHNNBFGK_06100 | phosphomethylpyrimidine synthase ThiC | primary metabolism |  | -1,89 |
| AHNNBFGK_06293 | RNA polymerase sigma factor RpoH | primary metabolism |  | -1,35 |
| AHNNBFGK_01858 | aminoacyl-tRNA hydrolase | protein expression |  | -4,87 |
| AHNNBFGK_04163 | gamma-glutamyl-gamma-aminobutyrate hydrolase family protein | putricine catabolism |  | 2,58 |
| AHNNBFGK_02072 | RNA pseudouridine synthase | RNA processing |  | 1,60 |
| AHNNBFGK_05037 | ribonuclease HII | RNA processing |  | 2,84 |
| AHNNBFGK_05179 | RNA helicase | RNA processing |  | 1,57 |
| AHNNBFGK_01149 | cobalamin biosynthesis protein | secondary metabolism |  | 1,86 |
| AHNNBFGK_02721 | type II toxin-antitoxin system ParD family antitoxin | secondary metabolism |  | -1,71 |
| AHNNBFGK_03147 | CobQ/CobB/MinD/ParA nucleotide binding domain-containing protein | secondary metabolism |  | -3,57 |
| AHNNBFGK_04049 | antibiotic biosynthesis monooxygenase | secondary metabolism |  | 2,00 |
| AHNNBFGK_02344 | response regulator | signal transduciton |  | -2,72 |
| AHNNBFGK_00550 | PAS domain S-box protein | signal transduction |  | 1,40 |
| AHNNBFGK_00942 | methyltransferase domain-containing protein | signal transduction |  | 2,39 |
| AHNNBFGK_01214 | class I SAM-dependent methyltransferase | signal transduction |  | -2,21 |
| AHNNBFGK_01538 | PAS domain S-box protein | signal transduction |  | 2,01 |
| AHNNBFGK_01557 | two-component sensor histidine kinase | signal transduction |  | -2,39 |
| AHNNBFGK_01892 | Methyltransferase | signal transduction |  | -2,09 |
| AHNNBFGK_01898 | PAS domain S-box protein | signal transduction |  | -1,98 |
| AHNNBFGK_02283 | class I SAM-dependent methyltransferase | signal transduction |  | 1,41 |
| AHNNBFGK_02697 | response regulator | signal transduction |  | 1,71 |
| AHNNBFGK_02754 | PAS domain S-box protein | signal transduction |  | 1,89 |
| AHNNBFGK_02862 | adenylate/guanylate cyclase domain-containing protein | signal transduction |  | 1,68 |
| AHNNBFGK_03307 | HAMP domain-containing histidine kinase | signal transduction |  | 3,55 |
|  |  |  | Continuation | |
|  |  |  |  |  |

| Genes | Description | Category | logFC 0,05 | logFC 0,50 |
| --- | --- | --- | --- | --- |
| AHNNBFGK_03964 | histidine kinase | signal transduction |  | 2,51 |
| AHNNBFGK_04592 | PrkA family serine protein kinase | signal transduction |  | -1,59 |
| AHNNBFGK_05275 | class I SAM-dependent methyltransferase | signal transduction |  | -4,16 |
| AHNNBFGK_05542 | response regulator | signal transduction |  | -3,52 |
| AHNNBFGK_05950 | response regulator | signal transduction |  | 1,55 |
| AHNNBFGK_05961 | HAMP domain-containing histidine kinase | signal transduction |  | -4,07 |
| AHNNBFGK_04061 | sulfurtransferase TusA family protein | sulfer metabolism |  | 1,57 |
| AHNNBFGK_00277 | sulfite exporter TauE/SafE family protein | transport |  | -3,05 |
| AHNNBFGK_00309 | TatABCE protein translocation system subunit (plasmid) | transport |  | -3,43 |
| AHNNBFGK_00461 | Trk system potassium transporter TrkA | transport |  | -2,41 |
| AHNNBFGK_00836 | ion transporter | transport |  | 1,51 |
| AHNNBFGK_00860 | MFS transporter | transport |  | 2,35 |
| AHNNBFGK_01145 | sulfite exporter TauE/SafE family protein | transport |  | 1,72 |
| AHNNBFGK_01340 | MFS transporter | transport |  | -2,76 |
| AHNNBFGK_01595 | amino acid ABC transporter permeasse | transport |  | -2,06 |
| AHNNBFGK_01889 | lysine transporter LysE | transport |  | -2,36 |
| AHNNBFGK_02047 | ABC transporter substrate-binding protein | transport |  | -2,00 |
| AHNNBFGK_02730 | TrkH family potassium uptake protein | transport |  | -1,94 |
| AHNNBFGK_02745 | DctP family TRAP transporter solute-binding subunit | transport |  | 1,91 |
| AHNNBFGK_02940 | manganese efflux pump | transport |  | -2,41 |
| AHNNBFGK_03005 | aspartate-alanine antiporter | transport |  | -1,48 |
| AHNNBFGK_03304 | C4-dicarboxylate ABC transporter permease | transport |  | 2,84 |
| AHNNBFGK_03658 | ABC transporter permease | transport |  | 1,89 |
| AHNNBFGK_04179 | branched-chain amino acid ABC transporter substrate-binding protein | transport |  | 2,24 |
| AHNNBFGK_04828 | ABC transporter ATP-binding protein | transport |  | 2,13 |
| AHNNBFGK_04919 | type VI secretion system protein TssA | transport |  | -1,86 |
| AHNNBFGK_06101 | oxalate/formate MFS antiporter | transport |  | -1,91 |
| AHNNBFGK_06225 | TRAP transporter permease | transport |  | -1,44 |
|  |  |  | Continuation | |
|  |  |  |  |  |
|  |  |  |  |  |
| Genes | Description | Category | logFC 0,05 | logFC 0,50 |
| AHNNBFGK_03561 | DUF2312 domain-containing protein | unknown |  | -1,97 |
| AHNNBFGK_05926 | DUF1328 domain-containing protein | unknown |  | -3,53 |
| AHNNBFGK_02652 | hypothetical protein | unknown |  | 1,63 |
| AHNNBFGK_03704 | hypothetical protein | unknown |  | -2,74 |
| AHNNBFGK_03705 | hypothetical protein TSH58_00630 | unknown |  | -3,38 |
| AHNNBFGK_05365 | hypothetical protein | unknown |  | 1,66 |
| AHNNBFGK_06256 | hypothetical protein | unknown |  | 1,88 |
| AHNNBFGK_02793 | right-handed parallel beta-helix repeat-containing protein | unknown |  | -1,78 |
|  |  |  | Conclusion | |

**
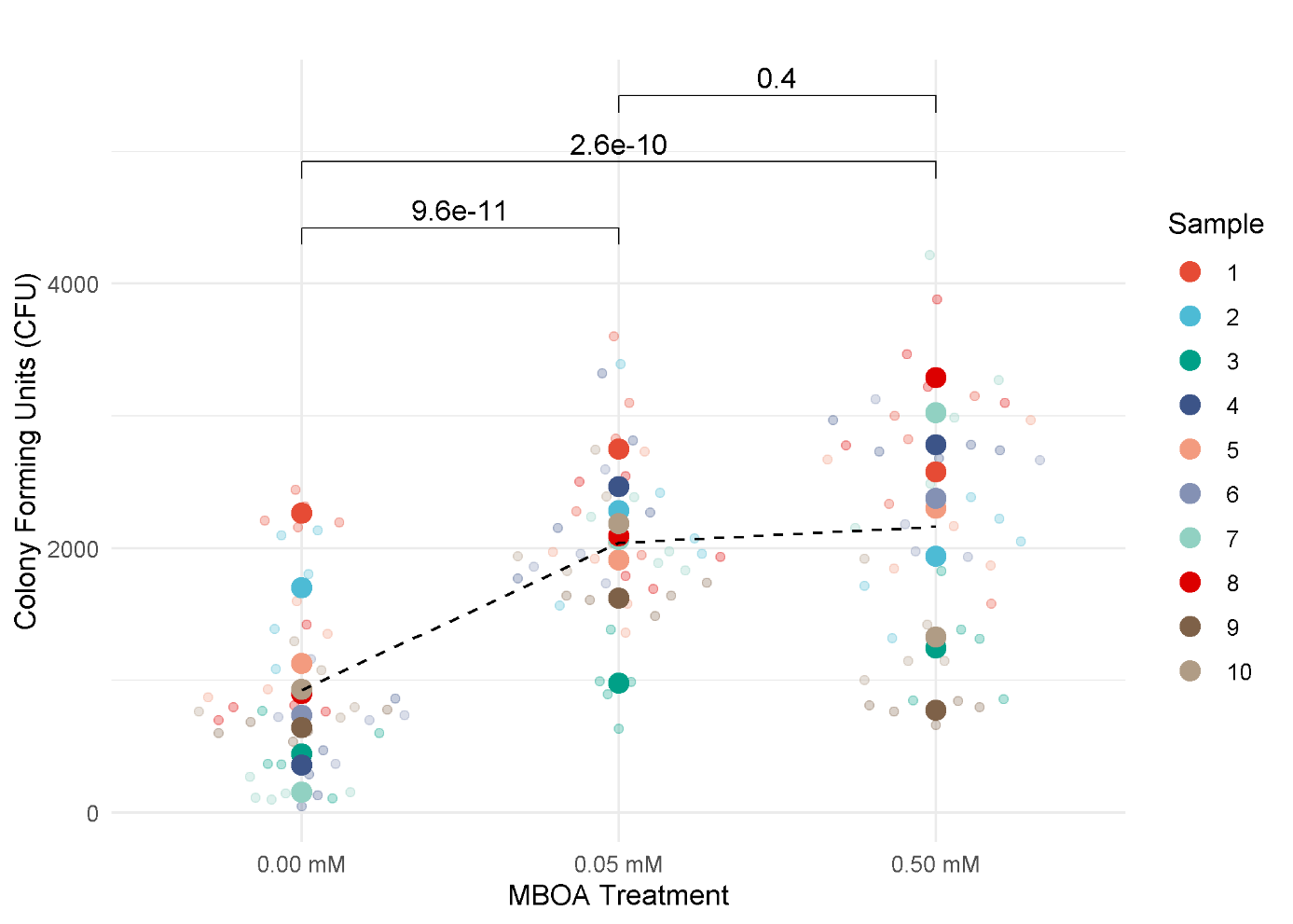
**

**Supplementary Figure 5**: Colony Forming Units (CFU) counted on DYGS agar plates by ImageJ via automated counting. Bacteria were collected after incubation for 15 minutes from syringes containing MBOA solution inserted in OD_600_ 0.05 *Azospirillum brasilense* Ab-V5 cultures. The superplot represent the mean values of ten biological replicates each with five technical replicates. P-values were calculated by a Wilcoxon Rank-Sum test.


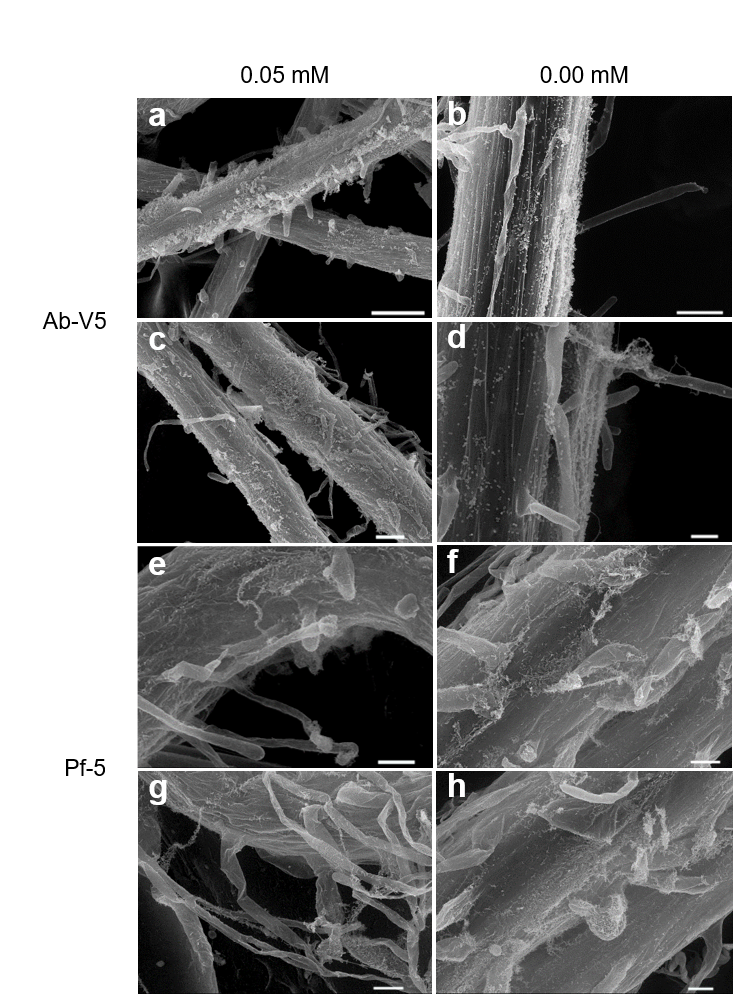


**Supplementary Figure 6**: Scanning electron microscopy of *Arabidopsis* roots inoculated with *Azospirillum brasilense* Ab-V5 and *Pseudomonas protegens* Pf-5. *A. thaliana* roots were inoculated 96 h before analysis with Ab-V5 (**a – d**) or Pf-5 (**e - h**) cultures of OD_600_ 0.05. **a, c, e** and **g**: 0.05 mM MBOA treatment. **b, d, f** and **h**: 0.00 mM MBOA treatment. Scale bars indicate 100 µm (**a**), 50 µm (**b, c**) and 20 µm (**d - h**).

#
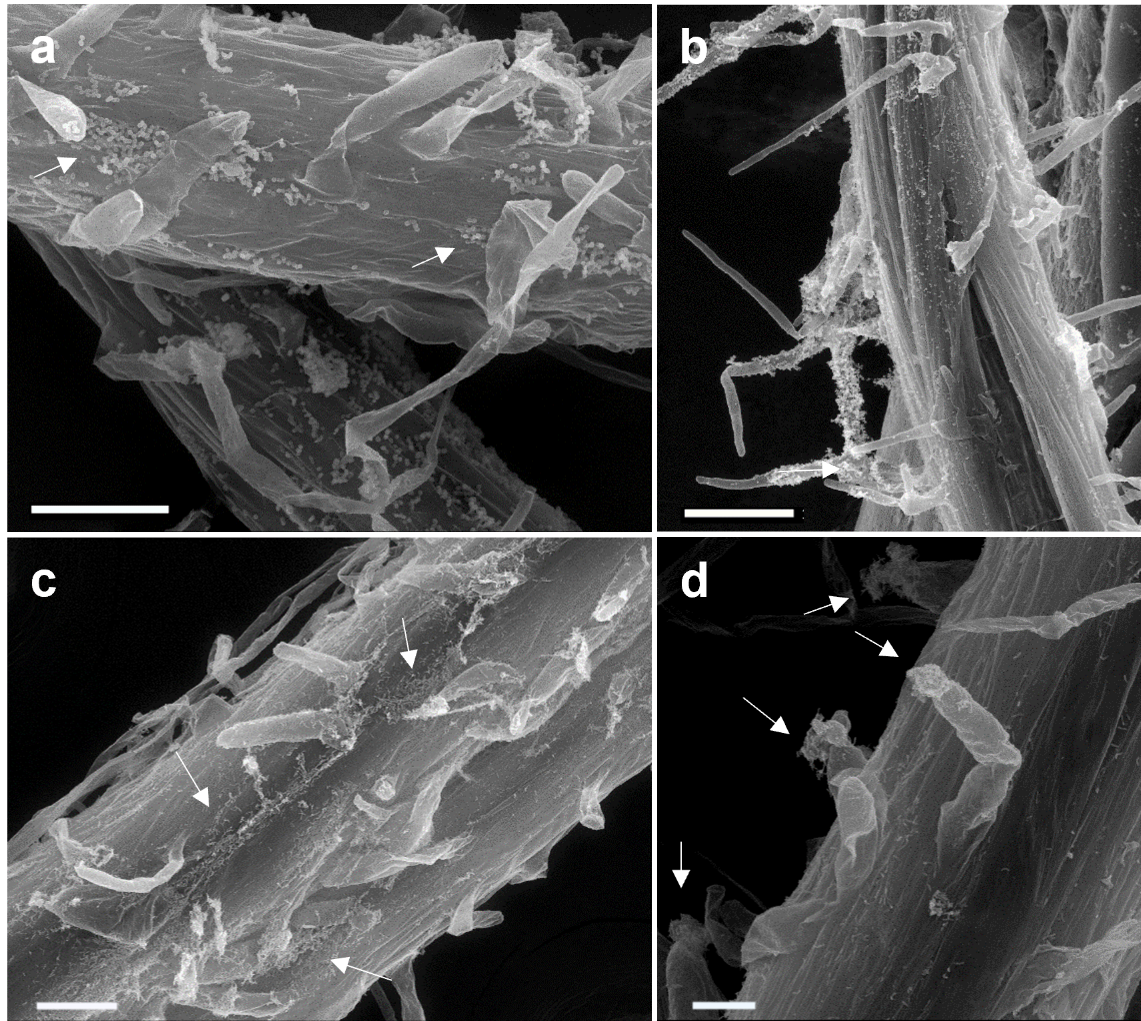


**Supplementary Figure 7**: Scanning electron microscopy of *Arabidopsis thaliana* Col-0 roots inoculated with *Azospirillum brasilense* Ab-V5 (**a, b**) and *Pseudomonas protegens* Pf-5 (**c, d**) of OD_600_ 0.5, 96 hours before the analysis. Arrows mark local accumulation of bacteria. Scale bars indicate 50 µm (**a, d**), 100 µm (**b, c, f**) and 20 µm (**e**).
